# Overexpression of Decorin Optimizes the Treatment Efficacy of Umbilical Cord Mesenchymal Stem Cells in Bleomycin-Induced Pulmonary Fibrosis in Rats

**DOI:** 10.1155/sci/6324980

**Published:** 2025-05-21

**Authors:** Yaofeng Zhi, Minghui Shu, Pinsheng Tang, Yingjie Li, Min Guo, Jiongrui Deng, Haixin Mo, Meimei Wu, Baoyi Liu, Yanyang Mai, Jie Ling, Xulin Zhao, Xin Zhang, Wanli Zuo

**Affiliations:** ^1^Clinical Experimental Center, Jiangmen Engineering Technology Research Center of Clinical Biobank and Translational Research, Jiangmen Key Laboratory of Precision and Clinical Translation Medicine, Jiangmen Central Hospital, Jiangmen, Guangdong, China; ^2^Department of Geriatric Medicine, The Affiliated Jiangmen Traditional Chinese Medicine Hospital of Jinan University, Jiangmen, Guangdong, China; ^3^Department of Cardiovascular, The Affiliated Jiangmen Traditional Chinese Medicine Hospital of Jinan University, Jiangmen, Guangdong, China; ^4^Department of Respiratory, The Affiliated Jiangmen Traditional Chinese Medicine Hospital of Jinan University, Jiangmen, Guangdong, China; ^5^Traditional Chinese Medicine Department, Guangdong Jiangmen Chinese Medicine College, Jiangmen, Guangdong, China; ^6^Department of Pulmonary and Critical Care Medicine, Jiangmen Central Hospital, Jiangmen, Guangdong, China; ^7^Dongguan Key Laboratory of Medical Bioactive Molecular Developmental and Translational Research, Guangdong Provincial Key Laboratory of Medical Molecular Diagnostics, Guangdong Medical University, Dongguan, Guangdong, China; ^8^Collaborative Innovation Center for Antitumor Active Substance Research and Development, Guangdong Medical University, Zhanjiang, Guangdong, China

**Keywords:** bleomycin, decorin, pulmonary fibrosis, umbilical cord mesenchymal stem cells

## Abstract

Idiopathic pulmonary fibrosis (IPF) is a long-term, diffuse pulmonary parenchyma lesion that primarily affects middle-aged and older adults. It is characterized by pulmonary interstitial fibrosis of unknown cause. The death rate upon diagnosis is higher than that of many other cancer types. Mesenchymal stem cell (MSC) treatment of organ fibrosis is a hot topic in preclinical and clinical research because it effectively treats IPF. In recent years, decorin (DCN) has been regarded as a critical mediator for its anti-inflammatory and antifibrotic effects. The purpose of this study was to generate human umbilical cord MSCs (HUC-MSCs) that overexpress DCN and to investigate the safety, mechanism, and effectiveness of using these cells to cure pulmonary fibrosis caused by bleomycin (BLM). First, lentiviral (LV) particles carrying the therapeutic DCN gene (LV-DCN) and control LV particles were created and transfected using the plasmid vector GV208 to create a viral solution for infecting HUC-MSCs. These solutions were used to create a DCN overexpression cell line and an MSC-Con. cell line infected with the control lentivirus. Intratracheal injection of BLM was used to establish a rat model of pulmonary fibrosis. On the second day following modeling, different treatments were administered, and the body weight and survival status of the rats were noted. The relevant tests were performed on days 15 and 29 following modeling. The results demonstrated that the overexpression of DCN did not affect the properties of HUC-MSCs and that these cells were effective in treating IPF. MSC-Con. and MSC-DCN reduced systemic inflammation by reducing serum interleukin (IL) 1*β*. Both cell types successfully treated pulmonary fibrosis in rats, as demonstrated by hematoxylin and eosin (HE) and Masson staining. MSC-DCN showed better efficacy due to lower mortality, higher weight gain, less alveolar inflammation, and less fibrosis. The safety of venous transplantation with MSCs was established by HE staining of the heart, liver, spleen, and kidney, as well as serum lactate dehydrogenase (LDH), creatinine (CRE), alanine aminotransferase (ALT), and aspartate aminotransferase (AST) levels. Immunohistochemical (IHC) staining of CD68 and CD206 in lung tissue and in vitro experiments on THP-1-induced M2 macrophage polarization and transforming growth factor-beta 1 (TGF-*β*1)-induced MRC-5 fibrosis indicated that MSC-DCN may mitigate lung inflammation by altering macrophage recruitment and polarization and inhibiting TGF-*β*1 expression to reduce fibrous hyperplasia and collagen deposition, thereby improving the treatment of BLM-induced IPF.

## 1. Introduction

Pulmonary fibrosis, with no age limit but mostly occurring in middle-aged and elderly patients, is one of the end-stage lung manifestations of various interstitial lung diseases that are represented by idiopathic pulmonary fibrosis (IPF) and is overwhelmingly accompanied by chronic diffuse pulmonary parenchymal lesions. The median survival time after diagnosis is ~3 years, and the mortality rate is higher than that in many types of cancer [1]. The pathogenesis of IPF has not been studied, and its treatment is limited. Currently, pirfenidone and nintedanib are approved for IPF treatment and can alleviate symptoms and slow further deterioration of the disease. Lung transplantation is the only treatment that can improve a patient's lifespan after diagnosis [2]. Mesenchymal stem cell (MSC) therapy may bring new hope for patients with IPF compared with the minimal effect of traditional treatment. However, the therapeutic effects of MSCs in clinical research remain controversial. This may be related to the time of the intervention, transplant dose, and cell quality [3]. MSCs from various sources have shown good efficacy in preclinical studies [2]. Clarifying the effects and molecular mechanisms of cell therapy on the occurrence and development of IPF is of great significance for improving clinical therapeutic effects and prognosis.

The treatment of IPF with MSCs is a hot topic in current research. MSCs, which can be extracted from the bone marrow, umbilical cord, or adipose tissue [4], can repair damaged tissue through their own or paracrine activity and mitochondrial transfer [5]. Our previous study showed that intravenous transplantation of MSCs from different sources reduced bleomycin (BLM)-induced lung tissue injury and delayed the progression of pulmonary fibrosis in rats, among which human umbilical cord MSCs (HUC-MSCs) had better curative effects. HUC-MSCs have a greater effect on the activation of regulatory T cells (Tregs) with immunomodulatory functions [6]. Although it can not completely simulate the characteristics of clinical IPF, the BLM-induced pulmonary fibrosis model is considered the cheapest, simplest, most reproducible, and most widely used animal model of IPF [7]. In recent years, our project team has committed to understanding the molecular mechanisms of and treating pulmonary fibrosis. Matrix metallopeptidase 9 (MMP9) and tissue metallopeptidase inhibitor 1 (TIMP1) regulate collagen deposition in the extracellular matrix (ECM) of the lungs, whereas high activity and imbalance of MMP9 and TIMP1 lead to collagen synthesis in the alveolar ECM and the occurrence and progression of pulmonary fibrosis. Early inhibition of MMP9 and TIMP1 in lung tissue and maintenance of balance between alveolar collagen synthesis and degradation are expected to become new strategies for the treatment of pulmonary fibrosis [8]. Circulating MMP9 and TIMP1 levels are generally elevated in patients with IPF [9]. Transforming growth factor beta (TGF-*β*) is a well-known mediator of pulmonary fibrosis [10]. Treatment with MSCs can downregulate TGF-*β* signaling and reduce the progression of fibrosis [11].

Decorin (DCN) is a leucine-rich proteoglycan that is ubiquitous in the ECM and can combine with collagen to inhibit the formation of collagen fibers and destroy the fiber structure [12]. DCN inhibits fibrosis after various tissue injuries by downregulating the TGF-*β* signaling pathway. Overexpression of DCN can block TGF-*β* signal transduction, reduce the production of other ECM components, and alleviate tissue fibrosis [13]. DCN has been widely used for the treatment of fibrotic diseases such as liver [14] and skin fibrosis [15]. In recent years, owing to the rapid development of gene editing technology, MSC function can be enhanced by gene modification, thereby providing a powerful treatment strategy for various stubborn diseases. Both DCN and HUC-MSCs have strong inhibitory effects on fibrosis, and studies have shown that DCN-modified MSCs have a positive effect on radiation-induced lung injury [16]. As tumor radiotherapy is the major cause of radiation-induced lung injury, we believe that the efficacy and safety of DCN-modified HUC-MSCs in the treatment of progressive pulmonary fibrosis is worthy of further study, considering the safety of MSCs in tumor-bearing patients. The purpose of this study was to demonstrate that DCN-modified HUC-MSCs are potentially more effective and safe for the treatment of lung fibrosis and to further explore its therapeutic dose and mechanism.

## 2. Materials and Methods

### 2.1. Animal Ethics

Seventy-two adult male Sprague-Dawley rats weighing ~200 ± 20 g were purchased from Zhuhai Bestest Bio-Tech (https://www.bestest100.com). They were kept in a specific pathogen-free (SPF) environment at the International Healthcare Innovation Institute (Jiangmen) at a constant temperature of 25°C, humidity range of 60%–70%, and 12 h of light per day, drinking and eating freely. All animal experiments were approved by the Experimental Animal Welfare and Ethics Committee of the department (N2022032). At the end of the experiment, blood was collected from the abdominal aorta under anesthesia with sodium pentobarbital (30 mg/kg), and the rats were exsanguinated under anesthesia from the abdominal aorta to euthanasia. All methods were performed in accordance with the relevant regulations and guidelines.

### 2.2. Plasmid Construction and Viral Solution Production

Using the commercial vector GV208, Genechem (https://www.genechem.com.cn/ Shanghai, China) was contracted to produce an overexpression plasmid for DCN (NM_001920). Next, 293T cells were transfected with a plasmid using polyethylenmine to produce a lentiviral (LV)-DCN viral solution at a titer of 3.5 + E9 transduction units/mL. The viral broth produced from the blank vector was designated as Con.

### 2.3. Cell Line Construction

Con. and LV-DCN virus solutions were mixed 1:100 (20 μL : 1980 μL) with stem cell serum-free medium (#NC0103+NC0103, Yocon, Beijing, China) and, used to infect HUC-MSCs (P1 cells, resuscitated 48 h prior and inoculated at 1 × 10^5^ cells/2 mL in 6-well plates), with three wells per group. HUC-MSCs were provided by CD Science, Ltd. (Guangzhou, China). After 10 h, the medium was replaced with fresh medium, and the cells were passaged when the degree of cell fusion reached 95%. The passage (P2) cells were cultured in a complete medium supplemented with 2 µL/mL puromycin (#HY-K1057, MCE, America). Cells that were not successfully transfected were screened for apoptosis. After one amplification passage, P4 MSC-Con. and MSC-DCN cell lines were established. A serum-free cell cryopreservation solution (#C40100, NCM Biotech. Suzhou, China) was used to cryopreserve the cells for further research.

### 2.4. Identification of DCN Expression

#### 2.4.1. Reverse Transcription-Quantitative Polymerase Chain Reaction (RT-qPCR)

Established MSC-Con. and MSC-DCN cell lines were cultured in 6-well plates at 1.2 × 10^5^ cells/2 mL/well for 72 h, with three wells per cell line. After 72 h, total RNA was extracted from the cells in every three wells. The cells were lysed with TRIzol (#R401-01, Vazyme Nanjing, China) to extract total RNA according to standard procedures [17]. Next, 1 µg total RNA was used to synthesize cDNA using a reverse transcription kit (#A0010CGQ, EZBioscience, shanghai, China). The forward and reverse primers for *DCN* and *β*-actin (DCN Forward: TGAGGGCTCCTGTGGCAAAT, Reverse: TGGCACTTTGTCCAGACCCAA. *β*-Actin, Forward: TGGATCAGCAAGCAGGAGTA, Reverse: TCGGCCACATTGTGAACTTT) were prepared by Shanghai Sangon Biotech according to the mRNA information from the National Center for Biotechnology Information. Color SYBR Green qPCR Master Mix (×2) (#A0012-R2, EZBioscience Shanghai, China) was used for RT-qPCR, and the data obtained (Gentier 96E, Tianlong, China) was analyzed according to standard processes and previously described methods [18].

#### 2.4.2. Western Blotting Analysis

MSC-Con. and MSC-DCN cultures were used for protein expression detection using anti-GAPDH (#60004-1-Ig, Proteintech, Wuhan, China) and DCN Polyclonal antibodies (#14667-1-AP, Proteintech, Wuhan, China) according to the standard operating procedure [19] and steps described in a previous study [20].

### 2.5. Flow Cytometry Identification of Stem Cell Surface Markers

MSC-Con. and MSC-CN cultures were subjected to cell digestion, and the following antibodies were incubated according to the manufacturer's instructions: anti-CD11b (CoraLite Plus 488 #CL488-65116), anti-CD19 (CoraLite Plus 488 #CL488-65197), CD34 (CoraLite Plus 488 #CL488−60180), CD73 (CoraLite Plus 405 #CL405-65162), CD90 (#66766-1-Ig), and CD105 (#10862-1-AP), with CD90 and CD105 reincubated with the secondary antibody Alexa Fluor405 (#ab175652, Abcam, Britain). Subsequently, flow cytometry was performed according to the standard procedures. Surface markers were selected based on the MSC minimum standards, and six were selected for detection [21, 22]. All antibodies were purchased from Proteintech (Wuhan, China) except for Alexa Fluor405. The machine used was Attune N-×T (Thermo, Massachusetts, America).

### 2.6. Apoptosis Assay

MSC-Con. and MSC-DCN cultures were subjected to cell digestion and incubated according to the AnnexinV-APC/PI Double-Staining Apoptosis Detection Kit (#KGA1107−100, Nanjing Jancheng Biotech, Nanjing, China) [20], and then detected using flow cytometry.

### 2.7. CCK-8 Assay

Transfected P6 MSC-Con. and MSC-DCN cell lines were inoculated into 96-well plates at 2 × 10^3^ cells/100 μL/well in 36 wells each. Next, 10 μL CCK-8 working solution (#FD3788-3000, Ford Biotech, Hangzhou, China) was added to six wells of each group on days 0, 1, 2, 3, 4, and 5. Cell proliferation was detected after incubation at 37°C for 1 h, and the absorbance was measured at 450 nm (Multiskan Fc, Thermo, America). The medium was replaced on day 3. Optical density values were measured daily and compared with those on day 0 to calculate the cell proliferation rate.

### 2.8. MSC Differentiation Assay

Transfected MSC-Con. and MSC-DCN were inoculated into 6 wells each of a 12-well plate at 8 × 10^4^ cells/1 mL/well. After 24 h, the two types of cells were replaced by an osteoblast differentiation induction culture system (#05465, Stemcell, Toronto, Canada) and an adipogenic differentiation induction culture system (#05412, Stemcell, Toronto, Canada), with three wells of each. The culture medium was changed every 3 days for the next 21 days. Subsequently, osteogenic differentiation, alizarin red staining, and adipogenic differentiation oil red O staining were performed as per standard procedures, and the stained images were captured under a microscope.

### 2.9. Cell Preparation

Established P4 MSC-Con. and MSC-DCN cell lines were resuscitated and cultured in a serum-free medium for 3 days. Cells were passaged 1:6 at ≥95% cell fusion, digested, collected, washed twice with saline, and counted after 3 days of culture. The cells were resuspended in 1 × 10^6^ or 5 × 10^6^ cells/500 μL/1.5 Eppendorf tube in saline and transported on ice to the SPF animal room within 1 h for rat tail vein transplantation.

### 2.10. Establishment and Treatment of BLM-Induced Pulmonary Fibrosis Model

The rats were fed for 3 days, anesthetized with sodium pentobarbital (30 mg/kg, i.p.), and the trachea was separated under aseptic conditions. BLM (Hanhui Pharmaceuticals Co., Shanghai, China) was injected into the trachea at 5 U/mL/kg, and the rats were rotated vertically from left to right for 1 min to ensure the BLM was evenly dispersed in the lungs. Finally, the incision was closed using sutures. About 24 h after modeling, the rats were randomly divided into the model, positive drug treatment (PA), MSC-Con., MSC-DCN Low and MSC-DCN High groups. The model group was administered 500 µL of saline via tail vein injection, and the PA group was treated with prednisone acetate (Zhongsheng Pharma, Dongguan, China) (3 mg/kg) by gavage for seven consecutive days. Our previous study found that comparing a high dose (10^7^) and low dose (10^6^) of BM-MSCs in the treatment of IPF model rats, the lung histopathology, degree of alveolitis, and degree of pulmonary fibrosis in the high-dose group were significantly better than those in the low-dose group. According to the official website of the China Drug Clinical Trial Registry (http://www.chinadrugtrials.org.cn/), the optimal dose for intravenous transplantation of MSCs for the treatment of IPF is between 3 × 10^7^ and 2 × 10^8^ cells. On this basis, two doses were chosen for the experiments. The MSC-Con. group was injected with 5 × 10^6^ cells of MSC-Con. The MSC-DCN Low group received 1 × 10^6^, and the MSC-DCN High group received 5 × 10^6^ MSC-DCN through the tail vein. The cells used for the injections were described as cell preparation contents. Half of the rats were euthanized on days 14 and 28 after treatment [23, 24], which was 15 and 29 days after modeling, to obtain the lung, spleen, kidney, liver, and heart tissues, as well as serum.

### 2.11. Blood Biochemical Assays

The sera obtained were subjected to serum MMP9 (#CSB-E08008r, CUSABIO, Wuhan, China), TIMP1 (#CSB-E08005r, CUSABIO, Wuhan, China), interleukin (IL) 1*β* (#ml028514, mlbio, Shanghai, China), IL10 (#ml028497, mlbio, Shanghai, China), superoxide dismutase (SOD) (#ml059387, mlbio, Shanghai, China) and malondialdehyde (MDA) (#ml077384, mlbio, Shanghai, China) tests, using enzyme-linked immunosorbent assays. Biochemical kits for serum lactate dehydrogenase (LDH) (#A020-2-2), creatinine (CRE) (#C011-2-1), alanine aminotransferase (ALT) (C009-2-1), and aspartate aminotransferase (AST) (C10-2-1) were obtained from the Nanjing Jiancheng Bioengineering Institute (Nanjing, China). The experimental procedures were performed according to the manual and published literature [25].

### 2.12. Histological Staining

After euthanasia, the lung, heart, spleen, kidney, and liver tissues of the rats were removed, paraffin-embedded, serially sectioned, and stained with hematoxylin and eosin (HE) and Masson staining according to standard pathology procedures. Images were obtained using the digital scanning microscopy imaging system on a microscope slide (PreciPoint M8, Germany). Referring to a previously published article [8], scores of 1, 2, 3, and 4 were assigned according to no, mild, moderate, and severe alveolar inflammation, respectively. Mild alveolitis is characterized by mononuclear cell infiltration, widened alveolar septa with normal structure, and infiltration confined to the proximal pleural area, involving <20% of the lungs. Moderate alveolitis involves 20%–50% of the lungs, and severe alveolitis involves >50% of the lung, with occasional mononuclear cells and hemorrhages in the alveolar cavities causing solid lesions. The degree of pulmonary fibrosis on Masson staining was scored according to the Ashcroft Score criteria [26]. Data are presented as median and interquartile range (M, [P25, P75]).

### 2.13. Immunohistochemical (IHC) Staining

IHC staining of the lung and spleen tissues was performed according to the same method used for histological staining and standard procedures to detect the expression of lung CD68 (#28058-1-AP), CD206 (#18704-1-AP), interferon-gamma (IFN-*γ*) (#15365-1-AP), tumor necrosis factor-alpha (TNF*α*) (#60291-1-Ig), TIMP1 (#16644-1-AP), MMP9 (#10375-2-AP), fibronectin-1 (FN1) (#66042-1-Ig), collagen type III (COL III) (#22734-1-AP), serum nuclear factor NF-E2-related factor (NRF2) (#80593-1-RR), quinone-forming reductase 1 (NQO1) (#67240-1-Ig), heme oxygenase-1 (HO1) (#66743-1-Ig), TGF-*β*1 (#21898-1-AP), and SMAD7 (#25840-1-AP), as well as the expression of spleen CD68 and CD206. All the antibodies were purchased from Proteintech (Wuhan, China). The experimental steps and expression scores for IHC were as described previously [27, 28]. The percentage of positive cells was graded as follows: 0, no positive cells; 1, <10% positive cells; 2, 10%–35% positive cells; 3, 35%–75% positive cells; and 4, >75% positive cells. Staining intensity was graded as follows: 0, no staining; 1, weak staining (yellowish); 2, moderate staining (yellowish-brown); 3, strong staining (brown) and 4, super strong staining (brown-black). The staining index was the product of the two scores. Staining indices in the different groups are presented as the median and interquartile range (M, [P25, P75]).

### 2.14. Cell Model Intervention Experiment In Vitro

In vitro experiments were performed to confirm the effects of MSC-DCN on macrophages and lung fibroblasts. The THP-1 and MRC-5 cell lines were purchased from Procell (Wuhan, China). TPH-1 cells were cultured in RPMI 1640 (#PM150110, Procell, Wuhan, Chian) supplemented with 10% fetal bovine serum (FBS) (#F102, Vazyme, Nanjing, China) and 1% penicillin–streptomycin solution (P/S) (#PB180120, Procell, Wuhan, China). MRC-5 cells were cultured in minimum essential medium (MEM) (#PM150410, Procell, Wuhan, China) supplemented with 10%FBS and 1% P/S. THP-1 and MRC-5 cells in the exponential growth phase were seeded in 12-well plates on glass coverslips. THP-1 cells were cultured with an additional 20 µg/L phorbol 12-myristate 13-acetate (PMA) (#P1585, Sigma–Aldrich, America) for 12 h to induce the M0 macrophage state, followed by 20 µg/L IL4 (#UA040026, UA. Bio, Hangzhou, Chian) and 20 µg/L IL13 (#UA040113, UA. Bio, Hangzhou, Chian) for 48 h to induce M2 macrophage polarization [29]. MRC-5 cells were cultured with 5 µg/L TGF-*β*1(#UA040085, UA. Bio, Hangzhou, Chian) and, after 24 h, seeded in 12-well plates for 48 h to induce the fibrogenic model. P4 MSC-Con. and MSC-DCN were cultured in a Transwell upper chamber in advance to 95% fusion and then transferred to the 12-well plates with THP-1 or MRC-5 cells at the bottom at the beginning of the cell model induction (Figure S7B,D). After 48 h of coculture, the expression of the target protein was detected by immunofluorescence according to the standard procedure and as previously described [30]. CD68 (#66231-2-Ig) and CD206 (#18704-1-AP) were detected in THP-1 cells whereas FN1 (#66042-1-Ig) and COL III (#22734-1-AP) were detected in MRC-5 cells. CD68 and FN1 were labeled with goat–anti-mouse secondary antibody (Alexa Fluor 488, #ab150113, Abcam), and CD206 and COL III were labeled with 647-conjugated goat–anti-rabbit secondary antibody (#RGAR005, Proteintech, Wuhan, China). DAPI was used to label the nuclei with an antifade mounting medium (#P0131, Beyotime, Shanghai, China). Images were acquired using a fluorescence microscope (Axio Imager M2, Ziss, Germany). The fluorescence intensity and number of nuclei were identified using ImageJ software. The ratio of fluorescence intensity to the number of nuclei represents the relative fluorescence intensity.

### 2.15. Data Analysis

All results are presented as the mean ± standard deviation or median and interquartile range (M, [P25, P75]). The corresponding data were statistically analyzed using GraphPad Prism 10 software. More than three groups were tested using Tukey's multiple comparisons test, and differences between the two groups were analyzed using an unpaired *t*-test. The log-rank (Mantel–Cox) test was used for survival analysis. Statistical significance was set at *p* ≤ 0.05.

## 3. Results

### 3.1. MSC-DCN Cell Line Was Successfully Generated Without Altering Its Stem Cell Properties

**Figure 1:**
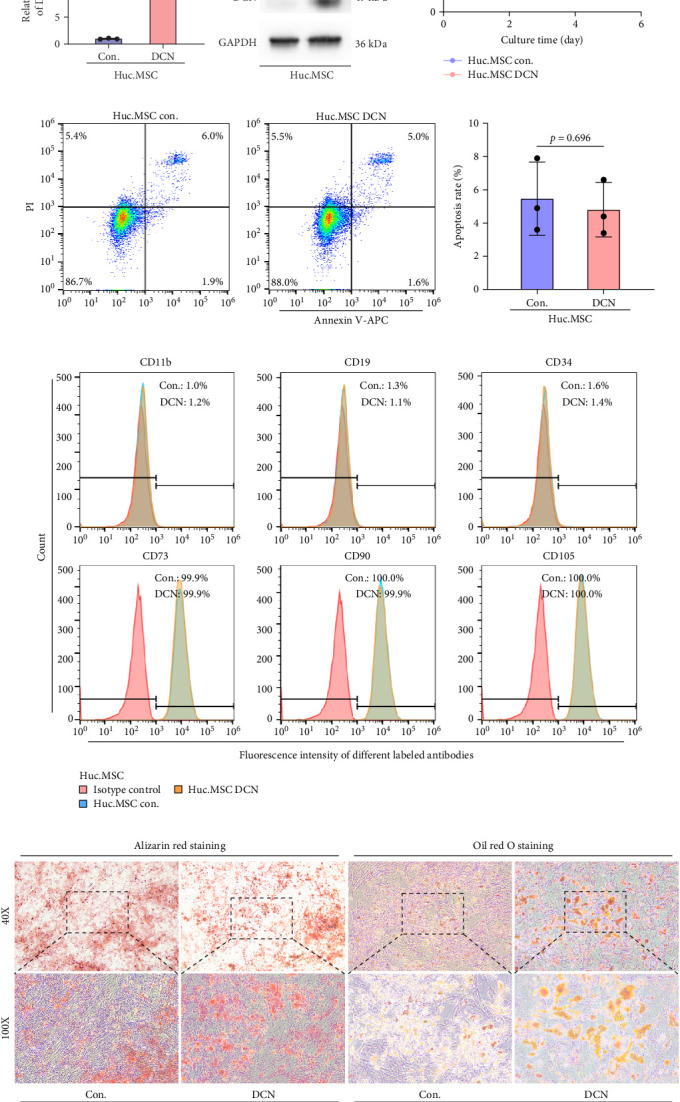
Decorin transfection does not effect the characteristics of MSC. (A) Expression of DCN in MSC after transfection. (B) DCN protein level in MSC after transfection. (C) Cell proliferation of MSC on days 0, 1, 2, 3, 4, and 5 after DCN overexpression. (D) Apoptosis of MSC after DCN overexpression. (E) MSC surface marker CD11b, CD19, CD34, CD73, CD90, and CD105 expression. (F) Alizarin red staining and oil red O staining after 21 days' differentiation of MSC. Each bar represents the mean ± SD (*n* = 3).

After LV infection and screening with puromycin (2 μg/mL), the MSC-Con. and MSC-DCN cell lines were generated. Compared to MSC-Con. the expression of the DCN gene and protein was significantly upregulated in MSC-DCN (Figure 1A,B). There were no surface markers changed of transfected MSCs, and they met the criteria for the identification of MSCs, with CD11b, CD19, and CD34 expressed <2%, whereas CD73, CD90, and CD105 were expressed >98% (Figure 1E). After the induction of osteogenic and adipogenic differentiation, the two groups of HUC-MSCs showed differentiation potential. After 21 days of osteogenic differentiation, alizarin red staining was positive, and calcified nodules were visible (Figure 1F). After 21 days of adipogenic differentiation, fat vesicle formation was observed using oil red O staining (Figure 1F). The CCK8 results showed that there was no difference in the proliferative ability of the two groups of cells, and they entered the exponential growth period when cultured for 48 h (Figure 1C). Flow cytometry after Annexin V-APC/PI staining showed that DCN overexpression did not promote apoptosis (Figure 1D). In conclusion, the MSC-DCN cell line was successfully constructed, and overexpression of the DCN protein did not affect the properties of MSCs.

### 3.2. Establishment of BLM-Induced Pulmonary Fibrosis Rat Model

On days 15 and 29 after the establishment of the model, half of the mice were anesthetized, and the corresponding organs were collected. About 4 days after BLM administration, all groups, except for the control group, gradually showed symptoms such as arched back, hard breathing, and mental burnout. The scatter plot of animal weight showed that the body weight of the model group was significantly lower than that of the control and PA groups (Figure S1A). Compared to the control group, the survival rates of the model and PA groups decreased significantly (Figure S1B), indicating that BLM led to the death of rats in the IPF model. On days 15 and 29, the lungs of rats in the model group showed severe alveolar inflammation and fibrosis, destruction of the alveolar structure, formation of cystic cavities, and other parenchymal lesions. Prednisone acetate showed therapeutic effects on day 29 of modeling after continuous gavage treatment and partially alleviated the symptoms of pulmonary fibrosis (Figure S1D,G). The scores for alveolar inflammation and fibrosis in the model group significantly increased, and PA treatment showed some efficacy in reducing the score 29 days after modeling (Figure S1E,F,H,I). Intratracheal administration had no effect on the rat heart, liver, kidney, and spleen structures (Figure S2A). There were no significant differences in the serum LDH, ALT, AST, or CRE levels; however, LDH, ALT, and AST levels increased slightly in the model group (Figure S2B–E). In addition, intragastric administration of prednisone acetate for 7 days increased the serum ALT levels in rats. Thus, we successfully established a rat model of BLM-induced pulmonary fibrosis. Prednisone acetate plays a role in alleviating the progression of pulmonary fibrosis; however, it generated side effects while exerting its action.

### 3.3. DCN Overexpression in HUC-MSC Can Be Used for Protecting BLM-Induced Pulmonary Fibrosis and Is Safe

**Figure 2:**
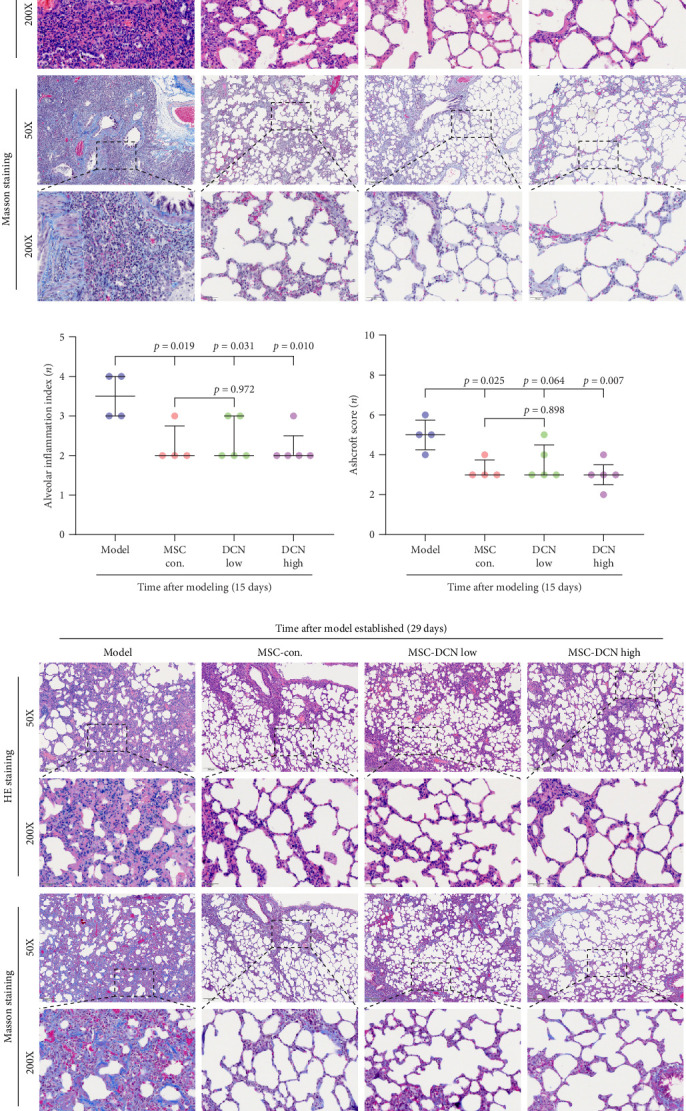
MSCs transplantation reduced bleomycin-induced pulmonary fibrosis in rats. (A) Schematic diagram of animal experiment. (B) Bodyweight of rats on days 15 and 29 after modeling. (C) HE and Masson staining in lung tissues on day 15 after modeling. (D) Alveolar inflammation index of lung tissues in rats on day 15 after modeling. (E) Ashcroft Score of lung tissues in rats on day 15 after modeling. (F) HE and Masson staining in lung tissues on day 29 after modeling. (G) Alveolar inflammation index of lung tissues in rats on day 29 after modeling. (H) Ashcroft Score of lung tissues in rats on day 29 after modeling. Alveolar inflammation index showed with scatter diagram, scoring 1, 2, 3, and 4 corresponding to no, mild, moderate, and severe alveolar inflammation. Scoring on Masson staining ranged 0–8 according to the Ashcroft Score criteria. Each bar represents the mean ± SD.

The day after the rats were intratracheally injected with BLM, they were subjected to tail vein transplantation of the corresponding cells, according to the MSC-Con., MSC-DCN Low, and MSC-DCN High groups. About 4 days after modeling, the rats in each group gradually showed symptoms, such as back-arching and hard breathing. Compared to the model group, MSC treatment gradually improved the symptoms. Death occurred in all groups, with the highest number of survivors in the MSC-DCN group; however, there was no statistically significant difference in the survival rate (Figure S1C). Compared with the model group, the mental state of rats recovered, and their body weights increased significantly after MSC-DCN and MSC-Con treatment, especially in the MSC-DCN High group. There was no difference in body weight between the MSC-Con. and MSC-DCN Low groups (Figure 2B). HE and Masson staining showed that HUC-MSC therapy reduced BLM-induced pulmonary fibrosis, including alveolar inflammation and pulmonary interstitial fibrosis (Figure 2C–H). No structural abnormalities were observed in the heart, liver, spleen, or kidneys (Figure S3A), and no differences were observed in serum LDH, AST, or CRE (Figure S3B,C,E) in each group treated with MSC. After 28 days of MSC treatment, the serum ALT levels in the other groups were lower than those in the model group, especially in the MSC-DCN High group (*p*=0.038, Figure S3D). As seen from the above results, DCN-overexpressing MSCs demonstrated better therapeutic potential in pulmonary fibrosis, as reflected by the fact that a smaller dose achieved a therapeutic effect roughly comparable to that of high-dose MSC-Con. The therapeutic effect of the same dose of MSC-DCN was superior to that of MSC-Con. Thus, HUC-MSC transplantation is a safe and effective treatment for pulmonary fibrosis.

### 3.4. HUC-MSC Overexpressing DCN Inhibits Inflammation and Fibrous Deposition in Lung Tissue

Based on these results, we concluded that MSC-Con. or MSC-DCN was effective in treating pulmonary fibrosis in rats. NQO1, HO1, and NRF2 are potent active components against tissue damage. We originally thought that MSC therapy could treat fibrosis by altering the local expression of NQO1, HO1, and NRF2 in lung tissues to play an anti-inflammatory and anticellular damage role. IHC results showed no difference in the expression of NQO1 among the three groups (Figure S4A,D). Compared with the control group, NRF2 in the model group was significantly upregulated after 29 days (Figure S4C), but there was no difference in expression between the PA and MSC transplantation groups compared to the model group (Figure S4F). Compared to the control group, the expression of HO1 in the model group was significantly upregulated at 15 and 29 days after modeling, and NRF2 in the lung tissue was significantly downregulated on the 29 days after PA treatment (Figure S4B,C). Compared with the model group, HO1 expression was significantly downregulated in the lungs of all groups treated with MSC transplantation, whereas there was no difference in the MSC-Con. and MSC-DCN Low groups (Figure S4E). There were no changes in the serum levels of SOD, a factor associated with antioxidant and anti-inflammatory damage, and subtle changes in MDA levels. There was no difference in the serum SOD content between the groups of rats on days 15 and 29 after modeling (Figure S4H,J). In contrast to the control group, the serum MDA content in the model group was significantly upregulated on day 15 after modeling, significantly decreased after PA treatment, and did not differ on day 29 after modeling (Figure S4G). Serum MDA was significantly downregulated in the MSC transplantation treatment groups on day 15 after modeling compared to the model group, while there was no difference in expression on day 29 (Figure S4I).

**Figure 3:**
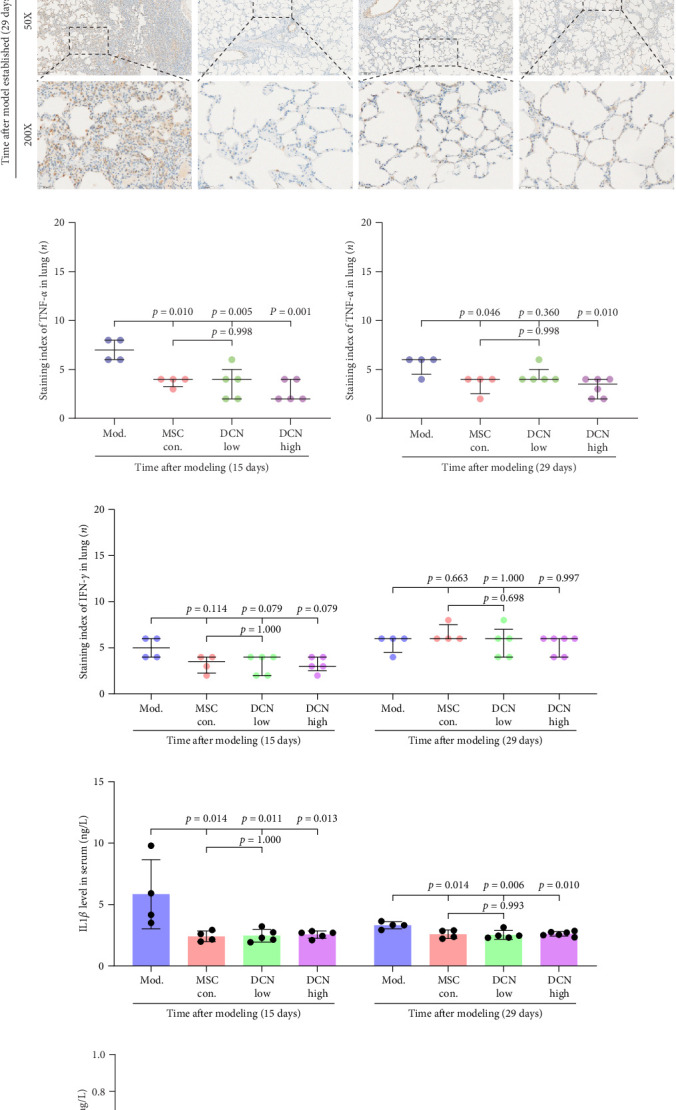
MSCs transplantation reduced the inflammation in Bleomycin-induced pulmonary fibrosis rats. (A) IHC staining of TNF-*α* in lung tissues on days 15 and 29 after modeling. (B) IHC staining index of TNF-*α* in lung tissues on day 15 after modeling. (C) IHC staining index of TNF-*α* in lung tissues on day 29 after modeling. (D) IHC staining index of IFN-*γ* in lung tissues on days 15 and 29 after modeling. (E) Serum IL1*β* levels. (F) Serum IL10 levels. Each bar represents the mean ± SD.

TNF*α* and IFN-*γ* respond to inflammatory levels in local tissues, whereas serum IL1*β* and IL10 respond to systemic inflammatory responses. Therefore, we detected serum IL1*β* and IL10 levels in rats on days 15 and 29 after modeling, respectively, as well as TNF*α* and IFN-*γ* levels in lung tissue using IHC. On days 15 and 29 after modeling, lung tissue TNF*α* expression was significantly upregulated in the model group compared to that in the control group, with a decreasing trend but no statistically significant difference after PA treatment (Figure S5A). IFN-*γ* was significantly upregulated in the model group at day 15 and tended to decrease in the PA group after treatment but was not statistically different, whereas there was no difference between all groups at day 29 (Figure S5B). Compared to the model group, both MSC-Con. and MSC-DCN treatments significantly downregulated the expression of tissue TNF*α* at both assay time points, and the results were almost the same between the MSC-DCN Low and the MSC-Con. groups (Figure 3A–C). However, IFN-*γ* levels were significantly different between the model and treatment groups (Figure 3D). Serum IL1*β* levels in rats in the model group increased significantly on day 15, whereas PA and normal levels appeared to be the same; however, there was no difference between the groups on day 29 (Figure S5C). Compared with the model group, the MSC treatment groups showed significantly reduced levels of IL1*β* (Figure 3E) on days 15 and 29. However, regardless of time, there was no significant difference in the serum IL10 levels among the groups (Figure 3F, Figure S5D). These results suggest that BLM can cause changes in the local and systemic inflammatory levels in rats, especially in the levels of the pro-inflammatory factor TNF*α* and serum IL1*β*. Stem cell therapy can significantly alleviate these symptoms. However, modeling and treatment had no effect on the anti-inflammatory factor IL10.

**Figure 4:**
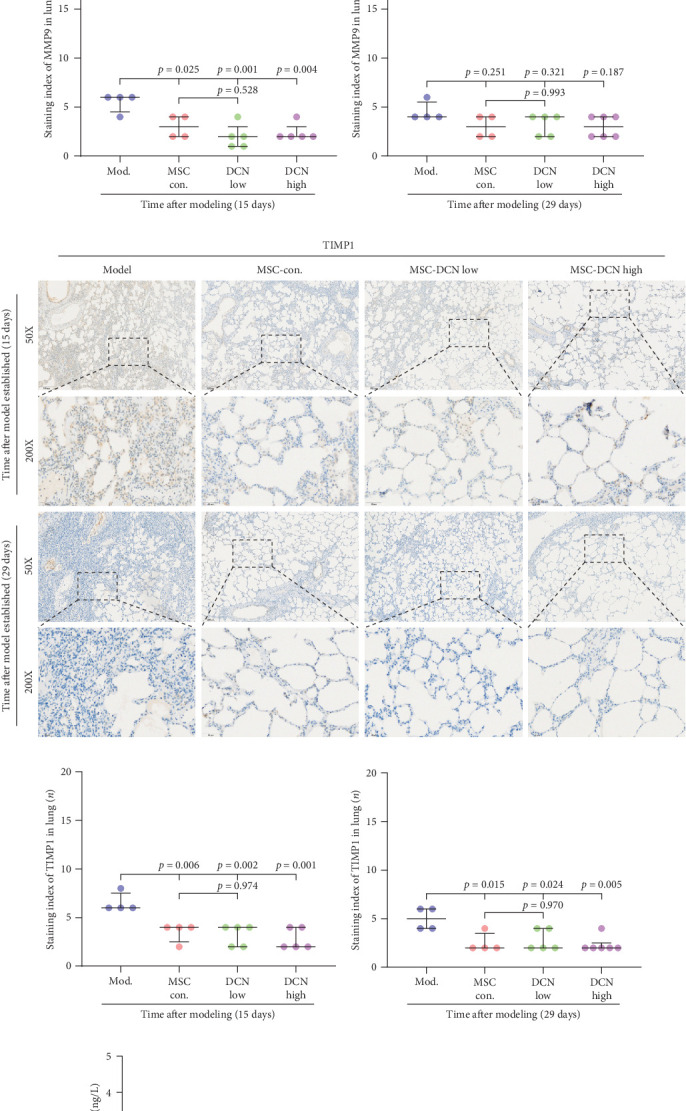
MSC transplantation effects the extracellular matrix remodeling in lung tissues. (A) IHC staining of MMP9 in lung tissues on days 15 and 29 after modeling. (B) IHC staining index of MMP9 in lung tissue on day 15 after modeling. (C) IHC staining index of MMP9 in lung tissue on day 29 after modeling. (D) IHC staining of TIMP1 in lung tissues on days 15 and 29 after modeling. (E) IHC staining index of TIMP1 on day 15 after modeling. (F) IHC staining index of TIMP1 on day 29 after modeling. (G) Serum MMP9 levels. (H) serum TIMP1 level. Each bar represents the mean ± SD.

MMP9 and TIMP1 are the main factors that maintain the balance between exudation and absorption of the local ECM. In the early stages of fibrosis, an increase in MMP9 levels leads to increased exudation of the local ECM and the production of raw materials for the subsequent formation of collagen. Compared to the control group, the expression of MMP9 and TIMP1 in the lung tissue of the model group increased significantly on days 15 and 29, and the expression of MMP9 decreased on 15 days after PA treatment (Figure S5E,F). High TIMP1 expression is likely due to self-repair feedback in rats. The expression of MMP9 was stable in the serum of rats in the control, model, and PA groups (Figure S5G) and was not affected by any experiment or treatment. The expression of serum TIMP1 was consistent with the normal levels found in lung tissue (Figure S5H). Ideal results could still be obtained. Compared with the model group, after 15 days of modeling, the MMP9 levels in the lung tissue of each cell treatment group were significantly reduced, whereas, at the 29-day time point, there was a downward trend without a statistically significant difference (Figure 4A–C). This may be because 29 days after modeling, the local tissue was already in the repair and collagen formation stages; therefore, the expression of MMP9 had stabilized, and the serum MMP9 level did not change significantly (Figure 4G). However, we still found that, compared with the model group, the tissue and serum levels of TIMP1 were significantly downregulated after MSC transplantation (Figure 4D–F,H), probably because rat fibrosis is significantly reversed after MSC transplantation, and thus, there is no need to produce excessive TIMP1 repair factor.

**Figure 5:**
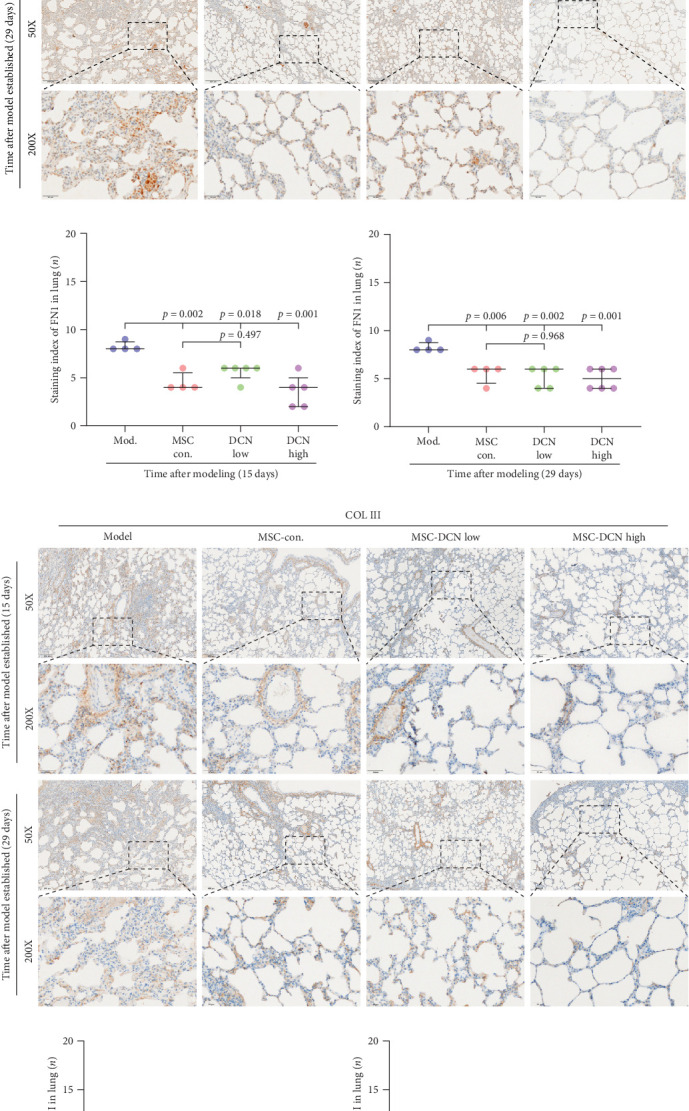
MSC transplantation reduced the local collagenous and fibrous deposition in lung tissues. (A) IHC staining of FN1 in lung tissues on days 15 and 29 after modeling. (B) IHC staining index of FN1 on day 15 after modeling. (C) IHC staining index of FN1 on day 29 after modeling. (D) IHC staining of COL III in lung tissues on days 15 and 29 after modeling. (E) IHC staining index of COL III on day 15 after modeling. (F) IHC staining index of COL III on day 29 after modeling.

The local expression levels of FN1 and COL III directly reflect the number of tissue' myofibroblasts and the degree of fiber deposition. IHC staining of FN1 and COL III in lung tissue showed that the expression of FN1 in the lung tissue of the model group increased significantly on days 15 and 29 compared with that in the control group, and PA treatment significantly improved this condition (Figure S5I). The expression of COL III in the model group also increased significantly, which significantly decreased in the early stage of PA treatment but did not show an effect on day 29 (Figure S5J). This further demonstrated that our experimental model was successful. Compared with the model group, FN1 and COL III in the lung tissue of the MSC treatment group were significantly reduced at both time points, and the downregulation effect in the MSC-DCN High group was more apparent (Figure 5A–F).

In summary, stem cells overexpressing DCN can improve the local cellular microenvironment in pulmonary fibrosis by regulating local and systemic inflammation levels and ultimately reducing fibrous deposition to achieve the effect of treating pulmonary fibrosis.

### 3.5. DCN Overexpression by MSCs Can Inhibit the Recruitment of Macrophages in Lung Tissue and Reduce M2 Polarization In Vitro

**Figure 6:**
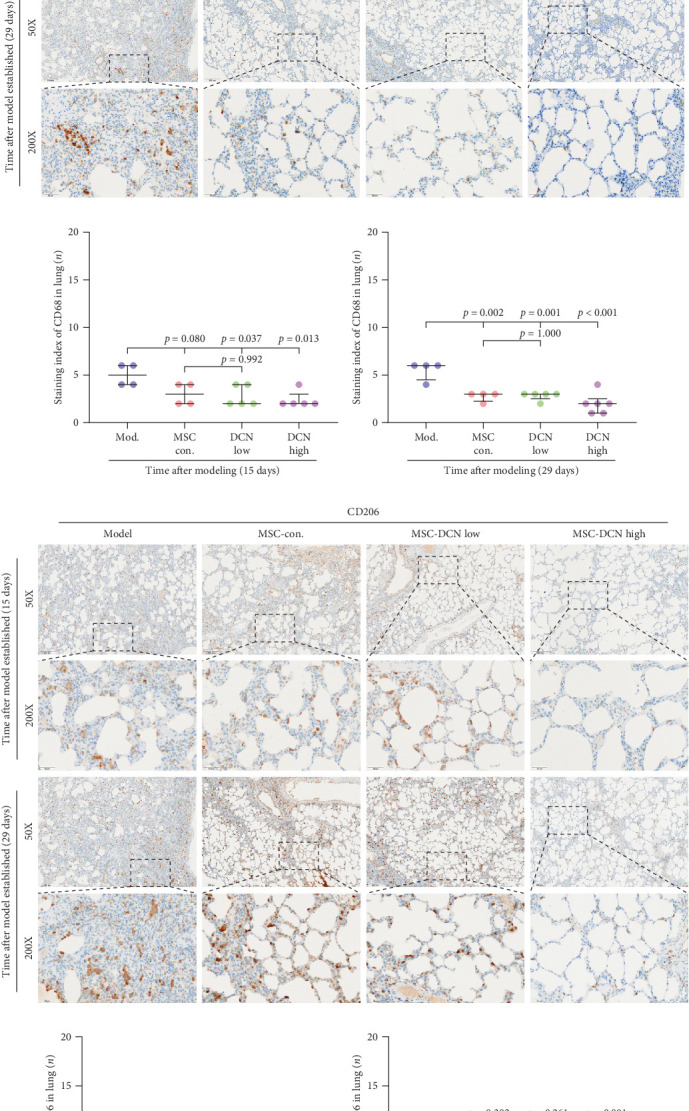
MSC transplantation reduced the macrophage recruitment and polarization in lung tissues. (A) IHC staining of CD68 in lung tissue on days 15 and 29 after modeling. (B) IHC staining index of CD68 on day 15 after modeling. (C) IHC staining index of CD68 on day 29 after modeling. (D) IHC staining of CD206 in lung tissue on days 15 and 29 after modeling. (E) IHC staining index of CD206 on day 15 after modeling. (F) IHC staining index of CD206 on day 29 after modeling.

Based on these results, we conclude that MSCs can effectively treat pulmonary fibrosis by improving systemic inflammation and suppressing the local inflammatory environment in lung tissue. We believe that this is related to macrophages. Macrophages are not only involved in local inflammation, but their phagocytic and secretory functions are also important for the formation of local fibrosis. As markers of macrophages and M2 macrophages, CD68 and CD206 not only reflect the recruitment of local macrophages but also distinguish their polarization. The IHC results showed that more macrophages were recruited to the lung tissue in the model group than in the normal group. Significantly more CD68 and CD206 positive macrophages were found in the lung tissues of the model group on days 15 and 29 after modeling (Figure S6A,B). PA treatment reduced the local recruitment of CD68 and CD206 positive cells in the later stages (Figure S6A,B). The immunomodulatory functions of MSCs are satisfactory. Intravenous transplantation has a lung-priming effect, with the majority of MSCs sequestered in the lungs. We found that local CD68-positive cells were significantly reduced in rat lungs in all cell treatment groups and that the MSC-DCN group was superior to the MSC-Con. group in reducing CD68-positive macrophage recruitment 15 days after modeling. The MSC-DCN High group showed the lowest CD68 positivity (Figure 6A–C). Conversely, CD206 expression was significantly reduced in the MSC-DCN High group, whereas no significant changes were observed in the MSC-Con. and MSC-DCN Low groups (Figure 6D–F). This suggests that DCN-overexpressing MSCs play a significant role in regulating macrophage polarization, which may be a key pathway for MSC treatment of pulmonary fibrosis.

**Figure 7:**
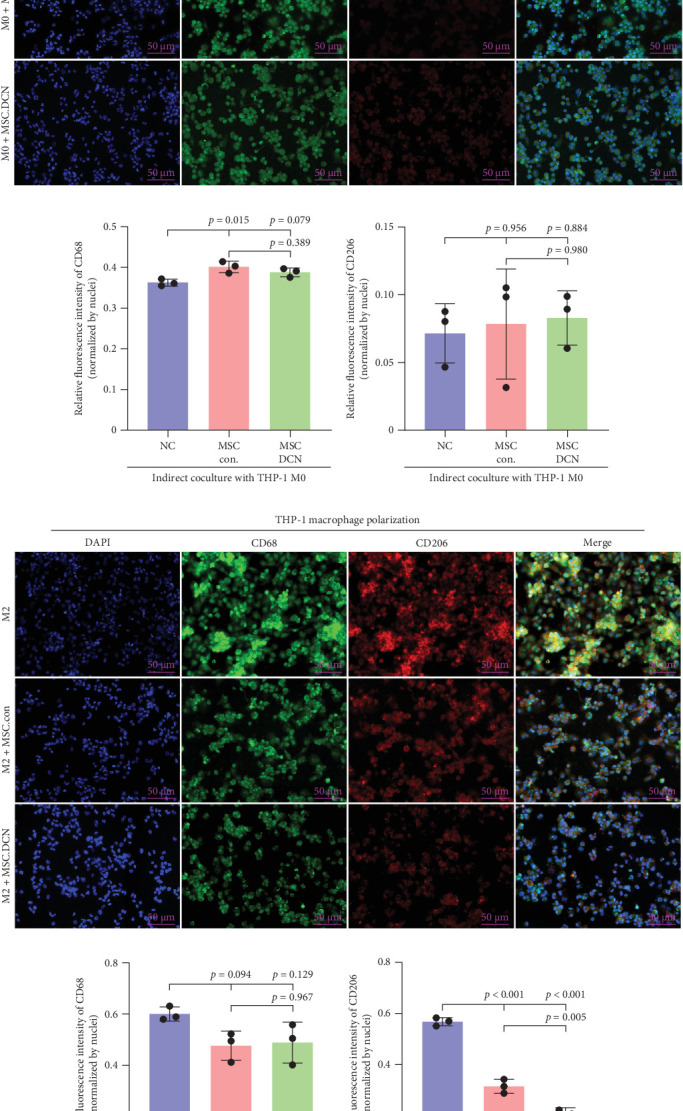
MSCs reduced the M2 macrophages polarization. (A) Immunofluorescence staining of CD68 and CD206 in THP-1-induced M0 macrophages. (B) Relative fluorescence intensity of CD68 in THP-1-induced M0 macrophages, compared to nuclei. (C) Relative fluorescence intensity of CD206 in THP-1-induced M0 macrophages, compared to nuclei. (D) Immunofluorescence staining of CD68 and CD206 in THP-1-induced M2 macrophages. (E) Relative fluorescence intensity of CD68 in THP-1-induced M2 macrophages, compared to nuclei. (F) Relative fluorescence intensity of CD206 in THP-1-induced M2 macrophages, compared to nuclei. Nuclei were costained with DAPI (blue). Each bar represents the mean ± SD (*n* = 3).

To further demonstrate the direct effect of MSCs on macrophage M2 polarization, we performed in vitro cell experiments. THP-1 cells were treated with PMA (185 µg/L) for 12 h to induce the M0 state and further treated with IL4 (20 µg/L) and IL13 (20 µg/L) for 48 h to induce the polarized M2 state (Figure S7A). Immunofluorescence of CD68 and CD206 showed that the coculture of MSCs and THP-1-induced M0 macrophages did not promote spontaneous M2 polarization (Figure 7A–C). However, upon progression to the M2 state, MSC coculture significantly reduced polarization, especially MSC with DCNs, which showed a greater effect (Figure 7D–F). IL10 levels in cell supernatants were also tested by flow cytometry. IL10 levels were noticeably increased after M2 induction and significantly decreased with MSC coculture (Figure S7C).

**Figure 8:**
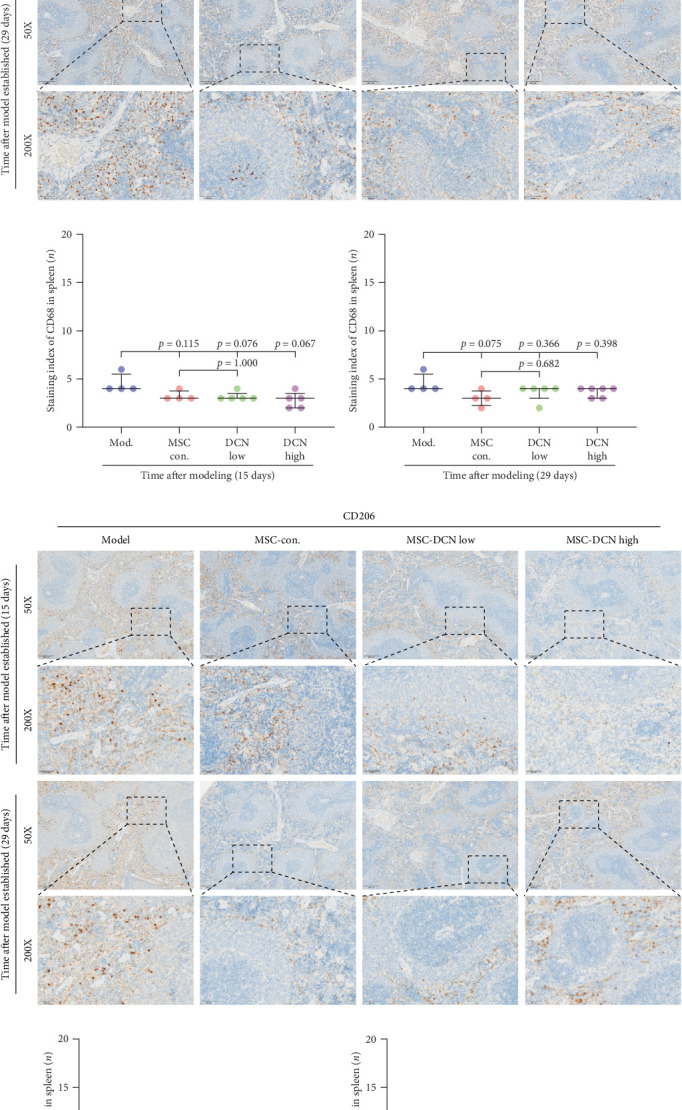
Spleen Macrophage recruitment after MSC transplantation. (A) IHC staining of CD68 in spleen tissues on days 15 and 29 after modeling. (B) IHC staining index of CD68 on day 15 after modeling. (C) IHC staining index of CD68 on day 29 after modeling. (D) IHC staining of CD206 in spleen tissues on days 15 and 29 after modeling. (E) IHC staining index of CD206 on day 15 after modeling. (F) IHC staining index of CD206 on day 29 after modeling.

The spleen is the main immune regulatory organ, and most macrophages originate from it. The number and polarization of splenic macrophages are feedback indices of systemic inflammation. After the establishment of the BLM model, the expression of CD68 and CD206 in the spleen showed an upward trend, but there was no statistically significant difference (Figure S6C,D). Although we observed a decrease in the expression of CD68 and CD206 in the spleens of rats treated with MSC transplantation, but this difference was not significant (Figure 8A–F). This may be due to an insufficient number of rats and an increase in the number of experimental animals show a difference.

### 3.6. Effect on TGF-*β* Signaling of MSC-DCN

**Figure 9:**
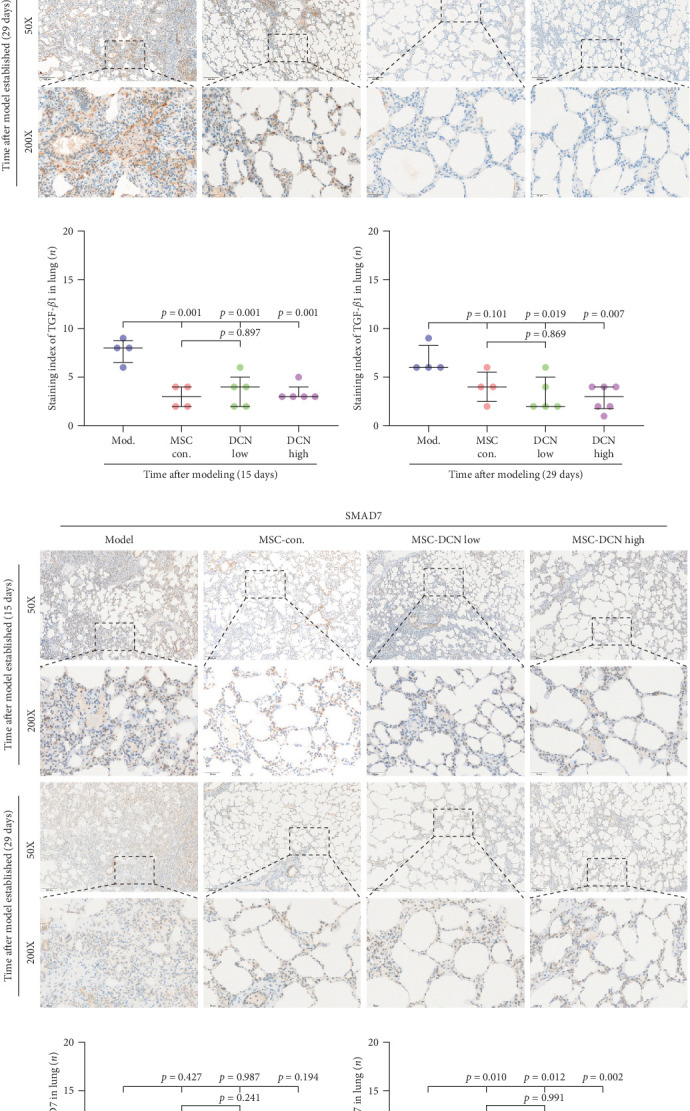
Effects of MSC-Con. and MSC-DCN on TGF-*β* signaling pathway. (A) IHC staining of TGF-*β*1 in lung tissues on days 15 and 29 after modeling. (B) IHC staining index of TGF-*β*1 on day 15 after modeling. (C) IHC staining index of TGF-*β*1 on day 29 after modeling. (D) IHC staining of Smad7 in lung tissues on days 15 and 29 after modeling. (E) IHC staining index of Smad7 on day 15 after modeling. (F) IHC staining index of Smad7 on day 29 after modeling.

The TGF-*β*/Smad signaling pathway is a key regulator of many fibrotic diseases. As described above, we successfully established a pulmonary fibrosis model. We found that TGF-*β*1 was significantly upregulated in all lung tissues of rats in the model group, and PA treatment significantly reduced TGF-*β*1 expression on day 15 after modeling; however, there was no statistically significant difference on day 29 (Figure S6E). In the cell transplantation treatment group, MSC-Con. and MSC-DCN showed a strong effect on reducing TGF-*β*1. TGF-*β*1 protein expression was downregulated in all cell transplantation groups, with the same effect observed in the MSC-Con. and MSC-DCN Low groups but more significantly in the MSC-DCN High group (Figure 9A–C). Smad7 acts as a negative regulator of TGF-*β* and was significantly downregulated in lung tissues in the model and PA groups (Figure S6F). After MSC treatment, local Smad7 expression was significantly increased in lung tissue on day 29 of modeling (Figure 9D–F). Thus, we demonstrated that MSCs play a role in the treatment of BLM-induced pulmonary fibrosis through the TGF-*β*1/Smad7 pathway and that MSCs overexpressing DCN showed better efficacy.

### 3.7. MSC-DCN Reduced TGF-*β*1-Induced Fibrosis in MRC-5 Cells

**Figure 10:**
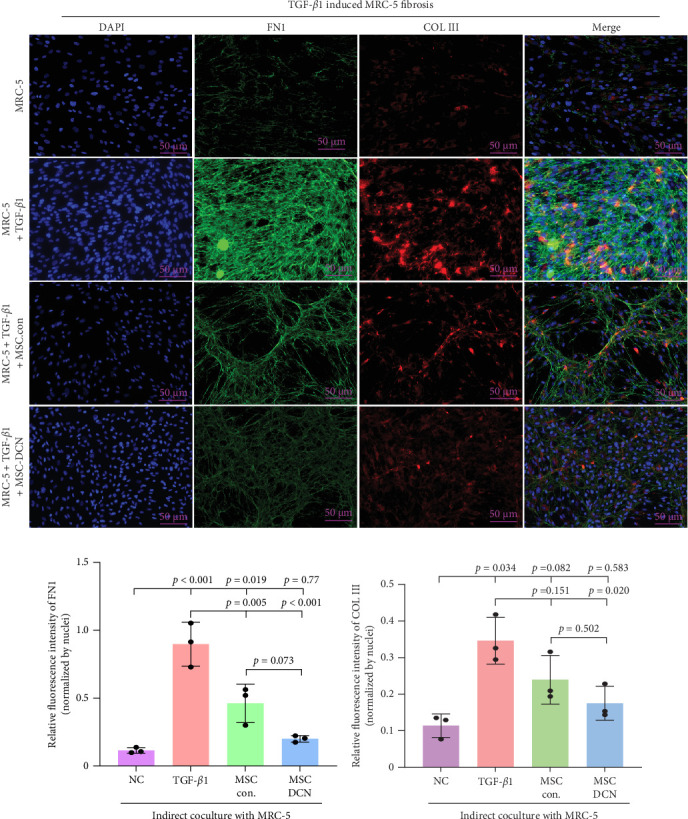
MSC-DCN reduced TGF-*β*1-induced fibrosis in MRC-5 cells. (A) Immunofluorescence staining of FN1 and COL III in MRC-5 cells. (B) Relative fluorescence intensity of FN1, compared to nuclei. (C) Relative fluorescence intensity of COL III, compared to nuclei. Nuclei were costained with DAPI (blue). Each bar represents the mean ± SD (*n* = 3).

To further demonstrate the effect of MSCs in reducing lung fibrosis, MRC-5 cells were treated with TGF-*β*1 to induce fibrosis. After TGF-*β*1 treatment for 48 h, the levels of the fibrosis-associated proteins FN1 and COL III in MRC-5 cells were considerably increased (Figure 10A–C). Coculture with MSCs significantly reduced the expression of FN1 and COL III in MRC-5 cells, with MSC-DCNs exhibiting a better effect (Figure 10A–C). Compared with the TGF-*β*1 group, COL III in the MSC-DCN cocultured group was significantly decreased, whereas the MSC-Con. group showed no statistical difference (Figure 10C).

## 4. Discussion

The pathogenesis of IPF remains unclear, and treatment options are limited. Currently, the main treatments are oral pirfenidone and nintedanib to relieve symptoms and slow further deterioration. Lung transplantation is the only treatment that can improve life expectancy after diagnosis [2]. BLM is one of the most commonly used drugs for inducing pulmonary fibrosis in animals. Intratracheal administration of BLM induces acute interstitial and alveolar inflammation and fibrotic changes in the trachea [31]. MSC therapy is considered a promising treatment for pulmonary fibrosis; however, its efficacy remains controversial. This may be related to the cell source, timing of the intervention, transplant dose, and cell quality [32]. Interestingly, MSCs have shown good efficacy in the early inflammatory stages of IPF and ultimately prevent the progression of pulmonary fibrosis [32]. Therefore, we selected the day after the tracheal administration of BLM as the treatment period. Our study demonstrated that MSCs overexpressing DCN can enhance the therapeutic effect on BLM-induced pulmonary fibrosis in rats by inhibiting inflammation and fibrosis deposition in the lung tissue and allowing rats to regain body weight. This effect was likely due to MSC-DCN reducing the recruitment of macrophages to the lung tissue. In addition, MSC-DCN can inhibit TGF-*β*1, a typical signaling pathway that promotes alveolar inflammation and fibrosis. Using blood indicators and organ morphology staining, we also confirmed that MSC therapy is not only effective but also safe. No side effects were observed during the experimental period, but prednisone acetate may have a certain effect on liver function.

Intratracheal injection of 5 U/mL/kg BLM, as described previously [33], successfully induced a rat model of pulmonary fibrosis. The damage was progressive and manifested as a large amount of inflammatory exudation, thickening of alveolar septa, and destruction of integrity. Later, the inflammatory environment could not be relieved and progressed to fibrous deposition, forming a cystic fibrous cavity, roughly as previously described [34, 35]. These symptoms are similar to those observed in interstitial pneumonia and pulmonary fibrosis [36].

Prednisone acetate has been studied for the treatment of diffuse fibrosing alveolitis [37], and oral prednisone can reduce BLM-induced collagen deposition in rats [38]. Currently, pirfenidone [39] and nintedanib [35] are mainly used to alleviate pulmonary fibrosis. For economic reasons, prednisone acetate is the preferred choice for some patients. Our results showed that continuous intragastric administration of prednisone acetate was effective against BLM-induced pulmonary fibrosis; however, its efficacy was not as good as that of MSC-Con. and MSC-DCN transplantation treatments. The symptoms of alveolar inflammation and fibrosis in rats in the PA group were reduced, as was the expression of FN1 and COL III in the lung tissue. However, local immune regulatory effects were not obvious. The effect of MSC transplantation was significantly greater than that observed in the PA group. MMPs and TIMPs play important roles in IPF pathogenesis [40]. The levels of MMP9 and TIMP1 in the alveolar lavage fluid of BLM-treated mice were increased [41], which is similar to that observed in patients with IPF [9]. The results of this study showed that MMP9 and TIMP1 expression were upregulated in the lung tissues of rats with BLM-induced pulmonary fibrosis. There was no change in the serum MMP9 levels, and TIMP1 was significantly upregulated. MSC treatment significantly reduced local and systemic TIMP1 levels, but the regulation of MMP9 in lung tissue had a significant effect only on day 15. This may be related to the early single transplantation of stem cells, as MSC have limited survival time in the body [42].

As described in our experimental results, MSC transplantation had a positive effect on the regulation of local lung tissue, systemic inflammation, and immune responses. When lung damage occurs, abnormally activated alveolar epithelial cells secrete higher levels of TGF-*β* and TNF-*α* [43]. These two factors promote fibroblast proliferation and the accumulation of ECM [31, 44]. The results of this study confirmed that MSC-Con. and MSC-DCN can significantly reduce the expression of TGF-*β* and TNF-*α* in lung tissue after treatment, and the reduction effect of MSC-DCN was more pronounced.

Patients with pulmonary fibrosis show IFN-*γ* activation due to the local accumulation of immune cells [45]. On day 15, after modeling, we detected an increase in IFN-*γ* in the lung tissue of the model group, and its expression tended to be stable between the model and treatment groups. On day 14 after MSC treatment, that is, 15 days after molding, there was a downward trend in IFN-*γ* in the lung tissues of rats treated with MSC-DCN (*p*=0.079). Some studies have indicated that the expression of IFN-*γ* in the serum of patients with progressive pulmonary fibrosis is higher than that in patients in the stable phase, especially during acute exacerbations of the disease [46]. After 2 weeks of modeling, the condition of the rats stabilized; therefore, the local expression of IFN-*γ* would also have stabilized. BLM-induced lung injury leads to IL-1*β* production and pulmonary fibrosis via activation of the NALP3 inflammasome [7]. The serum IL1*β* levels of the rats increased on day 15 after BLM modeling and decreased significantly after treatment with PA and MSCs. On day 29, after modeling, the serum IL1*β* of rats in the model group still showed an upward trend. Stem cell treatment significantly downregulated the IL1*β* level, whereas there was no downregulation effect in the PA group, demonstrating the anti-inflammatory ability of stem cells. The serum IL10 levels of the rats in each group remained stable and were not affected by modeling or drug intervention. The number of lymphocytes and granulocytes increase in the alveolar lavage fluid of IL10-specific deletion mice treated with BLM; however, there is no difference in lung tissue fibrosis from wild-type mice, suggesting that IL-10 plays a role in the immunomodulatory effects of alveolar inflammation but not in pulmonary fibrosis [47]. Sustained expression of IL-10 in lung tissue via an adeno-associated virus vector can ameliorate BLM-induced pulmonary fibrosis [48]. Fibroblast accumulation, collagen deposition, and ECM remodeling are hallmarks of IPF [49]. FN1 and COL III are the major components of the ECM and are produced by fibroblasts [50]. This study confirmed that the expression of FN1 and COL III was significantly upregulated in BLM-induced rat lung tissue and was significantly ameliorated after drug and cell treatment, among which high-dose MSC-DCN showed the most obvious effect. In vitro experiments have confirmed that hypoxia and oncostatin M preconditioned MSCs can alleviate TGF-*β*1-induced MRC-5 fibrosis [51, 52]. In our study, the coculture of MSC-Con. and MSC-DCN in transwells reduced TGF-*β*1-induced fibrosis by inhibiting the expression of FN1 and COL III, further demonstrating that MSCs can affect the fibrotic process via paracrine action.

Macrophages play an important role in lung homeostasis and tissue immune response after injury [7]. In the BLM-induced pulmonary fibrosis model, M1 macrophages increased rapidly, and M2 macrophages increased gradually. They reached their maximum value on day 14 and were positively correlated with the degree of fibrosis [53]. CD68^+^ M1 [54] and CD206^+^ M2 macrophages [55] can cause tissue fibrosis. As shown by IHC staining of lung tissue, CD68 and CD206 expression in the lung tissues of BLM-induced IPF rats was significantly upregulated. Regardless of whether MSCs overexpressed DCN, they significantly downregulated the expression of CD68, whereas only MSC-DCN at high doses significantly downregulated the expression of CD206. Therefore, we believe that MSC-DCN exerts a greater therapeutic effect by inhibiting the local accumulation of M2 macrophages in the lung tissue. Studies have confirmed that small molecule drugs, traditional Chinese medicine monomer combinations, and C/EBP homologous proteins inhibit the recruitment of local M2 macrophages to the lungs through different pathways and exhibit antifibrotic effects [56–58]. We also demonstrate that MSCs can inhibit THP-1 induced M2 macrophage polarization and that MSC-DCN showed better effect. The spleen is the largest immune organ in the human body. According to the IHC results, the tracheal administration of BLM did not increase splenic macrophage polarization. MSC transplantation also had a smaller impact on splenic macrophages but showed an overall downward trend. MSCs also regulate lung inflammation and damage by regulating the Th17/Treg ratio [59]. Numerous immune regulatory pathways for diseases exist, and our investigation has been limited in scope, having only conducted partial research. TGF-*β* is considered the most important component in fibrosis research. TGF-*β*1 produced by M2 macrophages can promote myofibroblast proliferation and collagen secretion, mediating fibrosis [7]. M2 macrophages secrete large amounts of TGF-*β* and induce epithelial-mesenchymal transition of alveolar epithelial cells in vitro through TGF-*β*-smad2 signaling [60]. Our study confirmed that MSC transplantation reduced the recruitment of M2 macrophages in lung tissue and significantly downregulated the expression of TGF-*β*1. Among them, the Smad7 positive ratio increased in rats treated with MSC-Con. and high does. Smad7 can negatively regulate TGF-*β*/Smad signaling by binding to TGF-*β* receptor Ⅱ to prevent Smad2/3 phosphorylation [61]. Several studies have reported that the upregulation of Smad7 is key to inhibiting TGF-*β* in the pathophysiology of fibrotic diseases and alleviating the progression of fibrosis [62, 63].

In addition, we performed IHC for NRF2, HO1, NQO1, and measured serum SOD and MDA levels. At the experimental time point, NRF2 and HO1 were upregulated in the lung tissues of rats with BLM-induced lung injury. HO1 levels were significantly reduced after treatment, whereas NRF2 levels showed no significant change. NQO1 showed an upward trend after modeling but showed no significant change after treatment. NRF2/HO1 has been considered antioxidative damage and antifibrosis signal in multiple studies and plays an important role in preventing myocardial [64], liver [65], lung [66], and kidney damage [67]. Without using compounds that act on this signaling pathway, it can be considered a damage-repair phenotype. Serum MDA levels were also significantly upregulated after BLM induction and significantly downregulated after treatment with PA or stem cells, indicating recovery and reduction of damage in rats after treatment. Serum SOD levels remained stable. The actual situation could potentially be reflected by measuring the content in alveolar lavage fluid.

## 5. Conclusion

In summary, our study confirmed that MSCs have a good therapeutic effect on BLM-induced lung injury and that MSCs overexpressing DCN have better therapeutic effects, including improved survival rate, reduced local inflammation, and fibrous deposition in the lung. This may be achieved by mediating the phenotype and recruitment of local macrophages, upregulating Smad7 expression, or reducing TGF-*β*1 expression. We successfully constructed DCN-modified MSCs that all the characteristics and proliferative abilities of stem cells and showed good results in animal treatment with high safety and efficacy but without side effects, providing practical evidence for clinical research on stem cell treatment of IPF.

However, this study had some limitations. Owing to the lack of a pulmonary function instrument, we did not detect the changes in the lung function of the rats. We only observed the physical condition of rats based on their survival rate, weight, and repetitive status. Thus, the experiment time point was chosen as the previous study. This instrument will be purchased to improve lung function detection in future studies. LV infection methods for constructing cell lines overexpressing genes also pose certain risks in clinical research. With advancements in CRISPR/Cas9 technology, the specific activation of DCN expression without genomic alteration may hold greater significance for clinical applications. However, further research is required in these areas.

## Data Availability

The data used to support the findings of this study are included within the article and Supporting Information figures.

## Supporting Information

Supporting Information Figure S1: (A) Bodyweight of rats on days 15 and 29 after modeling. (B) and (C) Survival after modeling and treatment. (D) HE and Masson staining in lung tissues on day 15 after modeling. (E) Alveolar inflammation index of lung tissues in rats on day 15 after modeling. (F) Ashcroft Score of lung tissues in rats on day 15 after modeling. (G) HE and Masson staining in lung tissues on day 29 after modeling. (H) Alveolar inflammation index of lung tissues in rats on day 29 after modeling. (I) Ashcroft Score of lung tissues in rats on day 29 after modeling. Alveolar inflammation index showed with scatter diagram, scoring 1, 2, 3, and 4 corresponding to no, mild, moderate, and severe alveolar inflammation. Scoring on Masson staining ranged 0–8 according to the Ashcroft Score criteria. Each bar represents the mean ± SD. Figure S2: (A) HE staining of heart, liver, kidney, and spleen. (B) Serum LDH levels. (C) Serum AST levels. (D) Serum ALT levels. (E) Serum CRE levels. Each bar represents the mean± SD. Figure S3: (A) HE staining of heart, liver, kidney, and spleen. (B) Serum LDH levels. (C) Serum AST levels. (D) Serum ALT levels. (E) Serum CRE levels. Each bar represents the mean± SD. Figure S4: (A) Immunohistochemical staining index of NQO1 in lung tissue on days 15 and 29 after modeling. (B) Immunohistochemical staining index of HO1 in lung tissue on days 15 and 29 after modeling. (C) Immunohistochemical staining index of NRF2 in lung tissue on days 15 and 29 after modeling. (D) Immunohistochemical staining index of NQO1 in lung tissue on days 15 and 29 after modeling. (E) Immunohistochemical staining index of HO1 in lung tissue on days 15 and 29 after modeling. (F) Immunohistochemical staining index of NRF2 in lung tissue on days 15 and 29 after modeling. (G) Serum MDA levels. (H) Serum SOD levels. (I) Serum MDA levels. (J) Serum SOD levels. Each bar represents the mean± SD. Figure S5: (A) Immunohistochemical staining index of TNF-*α* in lung tissue on days 15 and 29 after modeling. (B) Immunohistochemical staining index of IFN-*γ* in lung tissue on days 15 and 29 after modeling. (C) Serum IL1*β* levels. (D) Serum IL10 levels. (E) Immunohistochemical staining index of MMP9 in lung tissue on days 15 and 29 after modeling. (F) Immunohistochemical staining index of TIMP1 in lung tissue on days 15 and 29 after modeling. (G) Serum MMP9 levels. (H) Serum TIMP1 levels. (I) Immunohistochemical staining index of FN1 in lung tissue on days 15 and 29 after modeling. (J) Immunohistochemical staining index of COL III in lung tissue on days 15 and 29 after modeling. Each bar represents the mean± SD. Figure S6: (A) Immunohistochemical staining index of CD68 in lung tissue on days 15 and 29 after modeling. (B) Immunohistochemical staining index of CD206 in lung tissue on days 15 and 29 after modeling. (C) Immunohistochemical staining index of CD68 in spleen tissue on days 15 and 29 after modeling. (D) Immunohistochemical staining index of CD206 in spleen tissue on days 15 and 29 after modeling. (E) Immunohistochemical staining index of TGF-*β*1 in lung tissue on days 15 and 29 after modeling. (F) Immunohistochemical staining index of Smad7 in lung tissue on days 15 and 29 after modeling. Figure S7: (A) Different states of THP-1. (B) Schematic diagram of MSCs and THP-1 cells coculture. (C) IL10 levels in cell supernatant of different macrophage states. (D) Schematic diagram of MSCs and MRC-5 cells coculture. Each bar represents the mean ± SD (*n* = 3).

## References

[1] Liu Y. M., Nepali K., Liou J. P. (2017). Idiopathic Pulmonary Fibrosis: Current Status, Recent Progress, and Emerging Targets. *Journal of Medicinal Chemistry*.

[2] Spagnolo P., Kropski J. A., Jones M. G. (2021). Idiopathic Pulmonary Fibrosis: Disease Mechanisms and Drug Development. *Pharmacology and Therapeutics*.

[3] Tzouvelekis A., Toonkel R., Karampitsakos T. (2018). Mesenchymal Stem Cells for the Treatment of Idiopathic Pulmonary Fibrosis. *Frontiers in Medicine*.

[4] Wang Z., Chai C., Wang R. (2021). Single-Cell Transcriptome Atlas of Human Mesenchymal Stem Cells Exploring Cellular Heterogeneity. *Clinical and Translational Medicine*.

[5] Spees J. L., Lee R. H., Gregory C. A. (2016). Mechanisms of Mesenchymal Stem/Stromal Cell Function. *Stem Cell Research & Therapy*.

[6] Grégoire C., Ritacco C., Hannon M. (2019). Comparison of Mesenchymal Stromal Cells From Different Origins for the Treatment of Graft-vs.-Host-Disease in a Humanized Mouse Model. *Frontiers in Immunology*.

[7] Ishida Y., Kuninaka Y., Mukaida N., Kondo T. (2023). Immune Mechanisms of Pulmonary Fibrosis with Bleomycin. *International Journal of Molecular Sciences*.

[8] Zuo W. L., Zhao J. M., Huang J. X. (2017). Effect of Bosentan Is Correlated with MMP-9/TIMP-1 Ratio in Bleomycin-Induced Pulmonary Fibrosis. *Biomedical Reports*.

[9] Todd J. L., Vinisko R., Liu Y. (2020). Circulating Matrix Metalloproteinases and Tissue Metalloproteinase Inhibitors in Patients With Idiopathic Pulmonary Fibrosis in the Multicenter IPF-PRO Registry Cohort. *BMC Pulmonary Medicine*.

[10] Samarelli A. V., Tonelli R., Marchioni A. (2021). Fibrotic Idiopathic Interstitial Lung Disease: The Molecular and Cellular Key Players. *International Journal of Molecular Sciences*.

[11] Moodley Y., Vaghjiani V., Chan J. (2013). Anti-Inflammatory Effects of Adult Stem Cells in Sustained Lung Injury: A Comparative Study. *PLoS ONE*.

[12] Zhang W., Ge Y., Cheng Q., Zhang Q., Fang L., Zheng J. (2018). Decorin Is a Pivotal Effector in the Extracellular Matrix and Tumour Microenvironment. *Oncotarget*.

[13] Chaudhary K., Moore H., Tandon A., Gupta S., Khanna R., Mohan R. R. (2014). Nanotechnology and Adeno-Associated Virus-Based Decorin Gene Therapy Ameliorates Peritoneal Fibrosis. *American Journal of Physiology-Renal Physiology*.

[14] Jang Y. O., Cho M. Y., Yun C. O. (2016). Effect of Function-Enhanced Mesenchymal Stem Cells Infected With Decorin-Expressing Adenovirus on Hepatic Fibrosis. *Stem Cells Translational Medicine*.

[15] Järvinen T. A., Ruoslahti E. (2013). Targeted Antiscarring Therapy for Tissue Injuries. *Advances in Wound Care*.

[16] Liu D., Kong F., Yuan Y. (2018). Decorin-Modified Umbilical Cord Mesenchymal Stem Cells (MSCs) Attenuate Radiation-Induced Lung Injuries via Regulating Inflammation, Fibrotic Factors, and Immune Responses. *International Journal of Radiation Oncology∗Biology∗Physics*.

[17] Rio D. C., Ares M., Hannon G. J., Nilsen T. W. (2010). Purification of RNA Using TRIzol (TRI Reagent). *Cold Spring Harbor protocols*.

[18] Chen J., Liu A., Wang Z. (2020). LINC00173.v1 Promotes Angiogenesis and Progression of Lung Squamous Cell Carcinoma by Sponging miR-511-5p to Regulate VEGFA Expression. *Molecular Cancer*.

[19] Schägger H. (2006). Tricine–SDS-PAGE. *Nature Protocols*.

[20] Wu M. M., Wang Q. M., Huang B. Y. (2021). Dioscin Ameliorates Murine Ulcerative Colitis by Regulating Macrophage Polarization. *Pharmacological Research*.

[21] Troyer D. L., Weiss M. L. (2008). Wharton’s Jelly-Derived Cells Are a Primitive Stromal Cell Population. *Stem Cells*.

[22] Mushahary D., Spittler A., Kasper C., Weber V., Charwat V. (2018). Isolation, Cultivation, and Characterization of Human Mesenchymal Stem Cells. *Cytometry Part A: the Journal of the International Society for Analytical Cytology*.

[23] Wang Z., Li X., Chen H. (2021). Resveratrol Alleviates Bleomycin-Induced Pulmonary Fibrosis via Suppressing HIF-1*α* and NF-*κ*B Expression. *Aging*.

[24] Du W., Tang Z., Yang F., Liu X., Dong J. (2021). Icariin Attenuates Bleomycin-Induced Pulmonary Fibrosis by Targeting Hippo/YAP Pathway. *Biomedicine and Pharmacotherapy*.

[25] Wang C., Zhang L., Wu M. (2018). Antioxidative and Hepatoprotective Activities of the Ethyl Acetate Fraction Separated From the Fruit of *Livistona chinensis*. *Journal of Traditional Chinese Medicine (Chung i tsa chih ying wen pan)*.

[26] Ashcroft T., Simpson J. M., Timbrell V. (1988). Simple Method of Estimating Severity of Pulmonary Fibrosis on a Numerical Scale. *Journal of Clinical Pathology*.

[27] Zhang X., Zhang L., Lin B. (2017). Phospholipid Phosphatase 4 Promotes Proliferation and Tumorigenesis, and Activates Ca2+-Permeable Cationic Channel in Lung Carcinoma Cells. *Molecular Cancer*.

[28] Liu A., Zhang X., Li R. (2021). Overexpression of the SARS-CoV-2 Receptor ACE2 Is Induced by Cigarette Smoke in Bronchial and Alveolar Epithelia. *The Journal of Pathology*.

[29] Tedesco S., De Majo F., Kim J. (2018). Convenience Versus Biological Significance: Are PMA-Differentiated THP-1 Cells a Reliable Substitute for Blood-Derived Macrophages When Studying In Vitro Polarization?. *Frontiers in Pharmacology*.

[30] Mai C. T., Wu M. M., Wang C. L., Su Z. R., Cheng Y. Y., Zhang X. J. (2019). Palmatine Attenuated Dextran Sulfate Sodium (DSS)-Induced Colitis via Promoting Mitophagy-Mediated NLRP3 Inflammasome Inactivation. *Molecular Immunology*.

[31] Della Latta V., Cecchettini A., Del Ry S., Morales M. A. (2015). Bleomycin in the Setting of Lung Fibrosis Induction: From Biological Mechanisms to Counteractions. *Pharmacological Research*.

[32] Samarelli A. V., Tonelli R., Heijink I. (2021). Dissecting the Role of Mesenchymal Stem Cells in Idiopathic Pulmonary Fibrosis: Cause or Solution. *Frontiers in Pharmacology*.

[33] Zhao X., Wu J., Yuan R. (2023). Adipose-Derived Mesenchymal Stem Cell Therapy for Reverse Bleomycin-Induced Experimental Pulmonary Fibrosis. *Scientific Reports*.

[34] Moeller A., Ask K., Warburton D., Gauldie J., Kolb M. (2008). The Bleomycin Animal Model: A Useful Tool to Investigate Treatment Options for Idiopathic Pulmonary Fibrosis?. *The International Journal of Biochemistry and Cell Biology*.

[35] Richeldi L., Collard H. R., Jones M. G. (2017). Idiopathic Pulmonary Fibrosis. *The Lancet*.

[36] Lynch D. A., Travis W. D., Müller N. L. (2005). Idiopathic Interstitial Pneumonias: CT Features. *Radiology*.

[37] Labrune S., Chinet T., Collignon M. A., Barritault L., Huchon G. J. (1994). Mechanisms of Increased Epithelial Lung Clearance of DTPA in Diffuse Fibrosing Alveolitis. *European Respiratory Journal*.

[38] Shaker O. G., Sourour D. A. (2011). Effect of Leukotriene Receptor Antagonists on Lung Fibrosis in Rats. *Journal of Applied Toxicology*.

[39] Nathan S. D., Albera C., Bradford W. Z. (2017). Effect of Pirfenidone on Mortality: Pooled Analyses and Meta-Analyses of Clinical Trials in Idiopathic Pulmonary Fibrosis. *The Lancet Respiratory Medicine*.

[40] Pardo A., Cabrera S., Maldonado M., Selman M. (2016). Role of Matrix Metalloproteinases in the Pathogenesis of Idiopathic Pulmonary Fibrosis. *Respiratory Research*.

[41] Cinetto F., Ceccato J., Caputo I. (2021). GSK-3 Inhibition Modulates Metalloproteases in a Model of Lung Inflammation and Fibrosis. *Frontiers in Molecular Biosciences*.

[42] Kurtz A. (2008). Mesenchymal Stem Cell Delivery Routes and Fate. *International Journal of Stem Cells*.

[43] Drakopanagiotakis F., Wujak L., Wygrecka M., Markart P. (2018). Biomarkers in Idiopathic Pulmonary Fibrosis. *Matrix Biology*.

[44] Massagué J., Sheppard D. (2023). TGF-*β* Signaling in Health and Disease. *Cell*.

[45] Serezani A. P. M., Pascoalino B. D., Bazzano J. M. R. (2022). Multiplatform Single-Cell Analysis Identifies Immune Cell Types Enhanced in Pulmonary Fibrosis. *American Journal of Respiratory Cell and Molecular Biology*.

[46] Gui X., Qiu X., Tian Y. (2019). Prognostic Value of IFN-*γ*, sCD163, CCL2 and CXCL10 Involved in Acute Exacerbation of Idiopathic Pulmonary Fibrosis. *International Immunopharmacology*.

[47] Kradin R. L., Sakamoto H., Jain F., Zhao L. H., Hymowitz G., Preffer F. (2004). IL-10 Inhibits Inflammation but Does Not Affect Fibrosis in the Pulmonary Response to Bleomycin. *Experimental and Molecular Pathology*.

[48] Kurosaki F., Uchibori R., Sehara Y. (2018). AAV6-Mediated IL-10 Expression in the Lung Ameliorates Bleomycin-Induced Pulmonary Fibrosis in Mice. *Human Gene Therapy*.

[49] Chen X., Shi C., Meng X. (2016). Inhibition of Wnt/*β*-Catenin Signaling Suppresses Bleomycin-Induced Pulmonary Fibrosis by Attenuating the Expression of TGF-*β*1 and FGF-2. *Experimental and Molecular Pathology*.

[50] Kleaveland K. R., Moore B. B., Kim K. K. (2014). Paracrine Functions of Fibrocytes to Promote Lung Fibrosis. *Expert Review of Respiratory Medicine*.

[51] Lan Y. W., Choo K. B., Chen C. M. (2015). Hypoxia-Preconditioned Mesenchymal Stem Cells Attenuate Bleomycin-Induced Pulmonary Fibrosis. *Stem Cell Research and Therapy*.

[52] Lan Y. W., Theng S. M., Huang T. T. (2017). Oncostatin M-Preconditioned Mesenchymal Stem Cells Alleviate Bleomycin-Induced Pulmonary Fibrosis Through Paracrine Effects of the Hepatocyte Growth Factor. *Stem Cells Translational Medicine*.

[53] Ji W. J., Ma Y. Q., Zhou X. (2014). Temporal and Spatial Characterization of Mononuclear Phagocytes in Circulating, Lung Alveolar and Interstitial Compartments in a Mouse Model of Bleomycin-Induced Pulmonary Injury. *Journal of Immunological Methods*.

[54] Nakagawa M., Karim M. R., Izawa T., Kuwamura M., Yamate J. (2021). Immunophenotypical Characterization of M1/M2 Macrophages and Lymphocytes in Cisplatin-Induced Rat Progressive Renal Fibrosis. *Cells*.

[55] Wang J., Xu L., Xiang Z. (2020). Microcystin-LR Ameliorates Pulmonary Fibrosis via Modulating CD206+ M2-Like Macrophage Polarization. *Cell Death and Disease*.

[56] Ghebremedhin A., Salam A. B., Adu-Addai B. (2020). A Novel CD206 Targeting Peptide Inhibits Bleomycin Induced Pulmonary Fibrosis in Mice.

[57] Zhao P., Cai Z., Tian Y. (2021). Effective-Compound Combination Inhibits the M2-Like Polarization of Macrophages and Attenuates the Development of Pulmonary Fibrosis by Increasing Autophagy Through mTOR Signaling. *International Immunopharmacology*.

[58] Yao Y., Wang Y., Zhang Z. (2016). Chop Deficiency Protects Mice Against Bleomycin-Induced Pulmonary Fibrosis by Attenuating M2 Macrophage Production. *Molecular Therapy*.

[59] Chen J., Zhang X., Xie J. (2020). Overexpression of TGF*β*1 in Murine Mesenchymal Stem Cells Improves Lung Inflammation by Impacting the Th17/Treg Balance in LPS-Induced ARDS Mice. *Stem Cell Research and Therapy*.

[60] Zhu L., Fu X., Chen X., Han X., Dong P. (2017). M2 Macrophages Induce EMT Through the TGF-*β*/Smad2 Signaling Pathway. *Cell Biology International*.

[61] Hu H. H., Chen D. Q., Wang Y. N. (2018). New Insights Into TGF-*β*/Smad Signaling in Tissue Fibrosis. *Chemico-Biological Interactions*.

[62] Su D. N., Wu S. P., Xu S. Z. (2020). Mesenchymal Stem Cell-Based Smad7 Gene Therapy for Experimental Liver Cirrhosis. *Stem Cell Research and Therapy*.

[63] Yu J., Zhao X., Yan X. (2023). Aloe-Emodin Ameliorated MI-Induced Cardiac Remodeling in Mice via Inhibiting TGF-*β*/SMAD Signaling via up-Regulating SMAD7. *Phytomedicine: International Journal of Phytotherapy and Phytopharmacology*.

[64] Wu S., Zhu J., Wu G. (2022). 6-Gingerol Alleviates Ferroptosis and Inflammation of Diabetic Cardiomyopathy via the Nrf2/HO-1 Pathway. *Oxidative Medicine and Cellular Longevity*.

[65] Xu L., Yu Y., Sang R., Li J., Ge B., Zhang X. (2018). Protective Effects of Taraxasterol Against Ethanol-Induced Liver Injury by Regulating CYP2E1/Nrf2/HO-1 and NF-*κ*B Signaling Pathways in Mice. *Oxidative Medicine and Cellular Longevity*.

[66] Dang X., He B., Ning Q. (2020). Alantolactone Suppresses Inflammation, Apoptosis and Oxidative Stress in Cigarette Smoke-Induced Human Bronchial Epithelial Cells Through Activation of Nrf2/HO-1 and Inhibition of the NF-*κ*B Pathways. *Respiratory Research*.

[67] Lorestani F., Movahedian A., Mohammadalipour A., Hashemnia M., Aarabi M. H. (2024). Astaxanthin Prevents Nephrotoxicity Through Nrf2/HO-1 Pathway. *Canadian Journal of Physiology and Pharmacology*.

