# Supplementary material for: Overexpression of Decorin Optimizes the Treatment Efficacy of Umbilical Cord Mesenchymal Stem Cells in Bleomycin-Induced Pulmonary Fibrosis in Rats
Source: Stem Cells Int. 2025 May 21;2025:6324980. doi: 10.1155/sci/6324980 (PMC12119169; doi:10.1155/sci/6324980)
Supplement: Supporting Information — Figure S1: (A) Bodyweight of rats on days 15 and 29 after modeling. (B) and (C) Survival after modeling and treatment. (D) HE and Masson staining in lung tissues on day 15 after modeling. (E) Alveolar inflammation index of lung tissues in rats on day 15 after modeling. (F) Ashcroft Score of lung tissues in rats on day 15 after modeling. (G) HE and Masson staining in lung tissues on day 29 after modeling. (H) Alveolar inflammation index of lung tissues in rats on day 29 after modeling. (I) Ashcroft Score of lung tissues in rats on day 29 after modeling. Alveolar inflammation index showed with scatter diagram, scoring 1, 2, 3, and 4 corresponding to no, mild, moderate, and severe alveolar inflammation. Scoring on Masson staining ranged 0–8 according to the Ashcroft Score criteria. Each bar represents the mean ± SD. Figure S2: (A) HE staining of heart, liver, kidney, and spleen. (B) Serum LDH levels. (C) Serum AST levels. (D) Serum ALT levels. (E) Serum CRE levels. Each bar represents the mean± SD. Figure S3: (A) HE staining of heart, liver, kidney, and spleen. (B) Serum LDH levels. (C) Serum AST levels. (D) Serum ALT levels. (E) Serum CRE levels. Each bar represents the mean± SD. Figure S4: (A) Immunohistochemical staining index of NQO1 in lung tissue on days 15 and 29 after modeling. (B) Immunohistochemical staining index of HO1 in lung tissue on days 15 and 29 after modeling. (C) Immunohistochemical staining index of NRF2 in lung tissue on days 15 and 29 after modeling. (D) Immunohistochemical staining index of NQO1 in lung tissue on days 15 and 29 after modeling. (E) Immunohistochemical staining index of HO1 in lung tissue on days 15 and 29 after modeling. (F) Immunohistochemical staining index of NRF2 in lung tissue on days 15 and 29 after modeling. (G) Serum MDA levels. (H) Serum SOD levels. (I) Serum MDA levels. (J) Serum SOD levels. Each bar represents the mean± SD. Figure S5: (A) Immunohistochemical staining index of TNF-α in lung tissue on days 15 and 29 afte [file 6324980.f1.pdf]

Supplemental figure 1

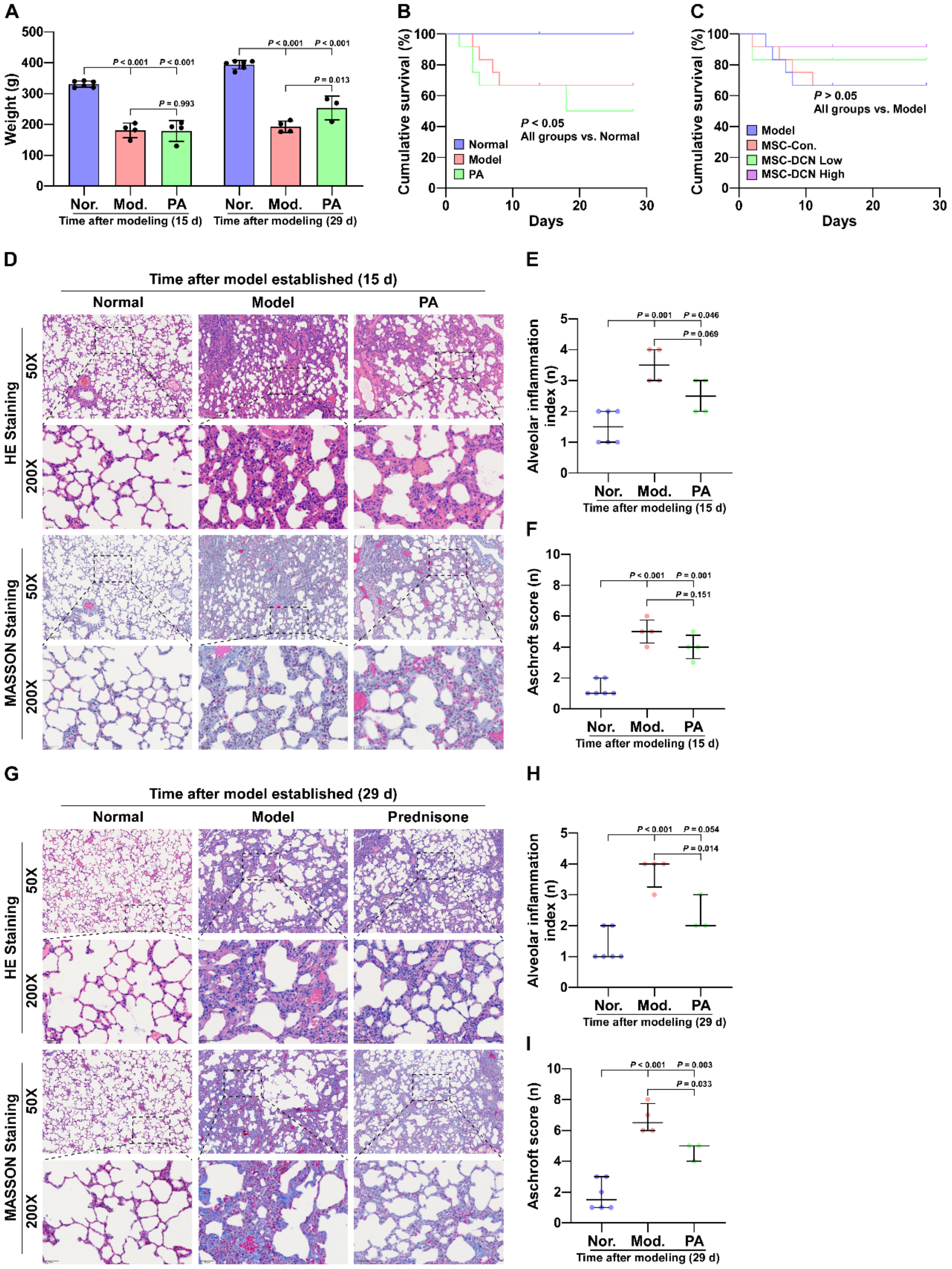

Supplemental figure 1. Establishment of bleomycin-induced pulmonary fibrosis rat model. (A) Body wight of rats on days 15 and 29 after modelling. (B) and (C) Survival after modelling and treatment. (D) HE and Masson staining in lung tissues on day 15 after modelling. (E) Alveolar inflammation index of lung tissues in rats on day 15 after modelling. (F) Aschroft Score of lung tissues in rats on day 15 after modelling. (G) HE and Masson staining in lung tissues on day 29 after modelling. (H) Alveolar inflammation index of lung tissues in rats on day 29 after modelling. (I) Aschroft Score of lung tissues in rats on day 29 after modelling. Alveolar

inflammation index showed with scatter diagram, scoring 1, 2, 3, and 4 corresponding to no, mild, moderate, and severe alveolar inflammation. Scoring on Masson staining ranged 0 to 8 according to the Aschroft Score criteria. Each bar represents the mean ± SD.

Supplemental figure 2

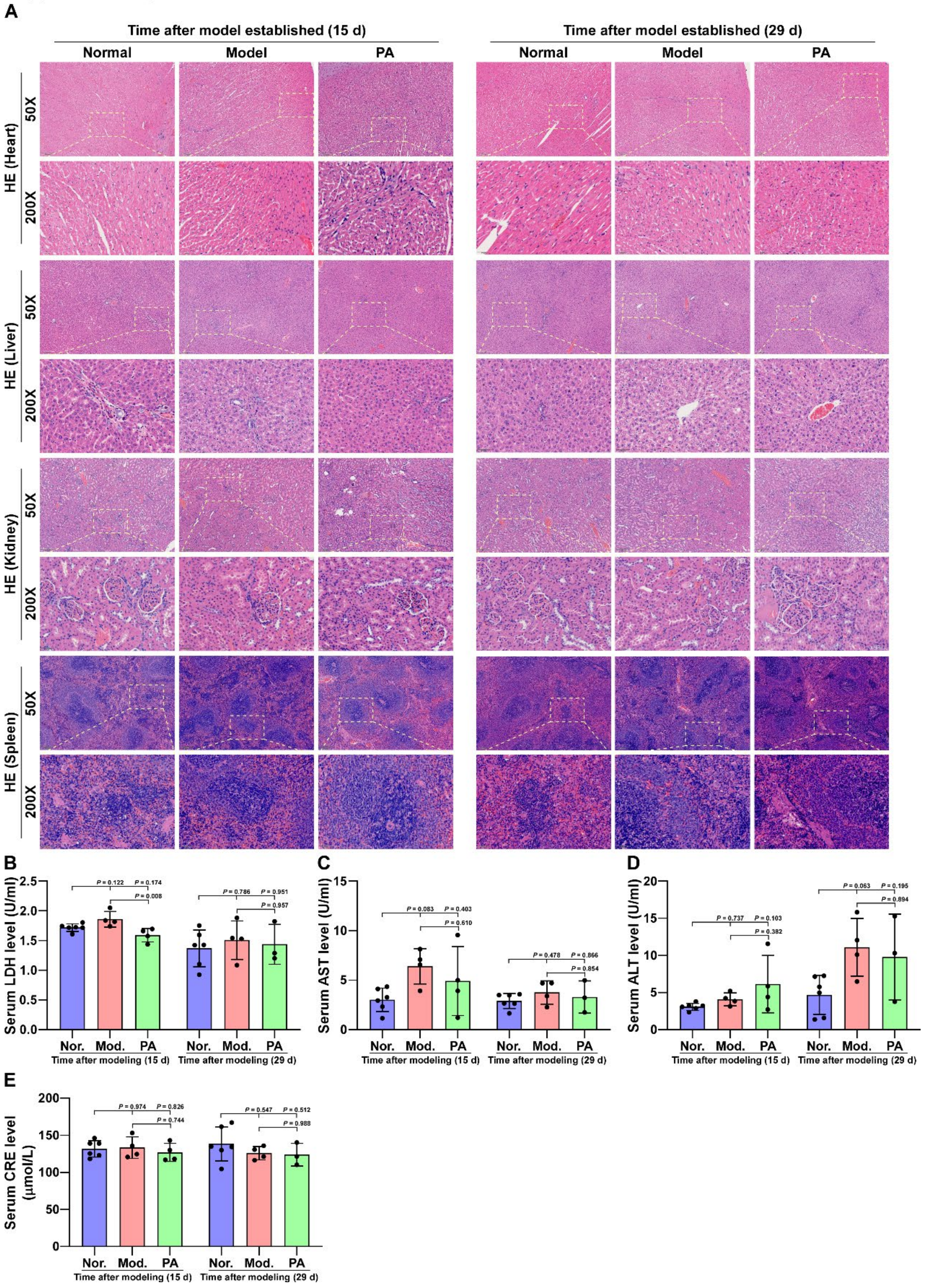

Supplemental figure 2. General reaction of Bleomycin-induced pulmonary fibrosis rat model. (A) HE staining of heart, liver, kidney and spleen. (B) Serum LDH levels. (C) Serum AST levels. (D) Serum ALT levels. (E) Serum CRE levels. Each bar represents the mean± SD.

Supplemental figure 3

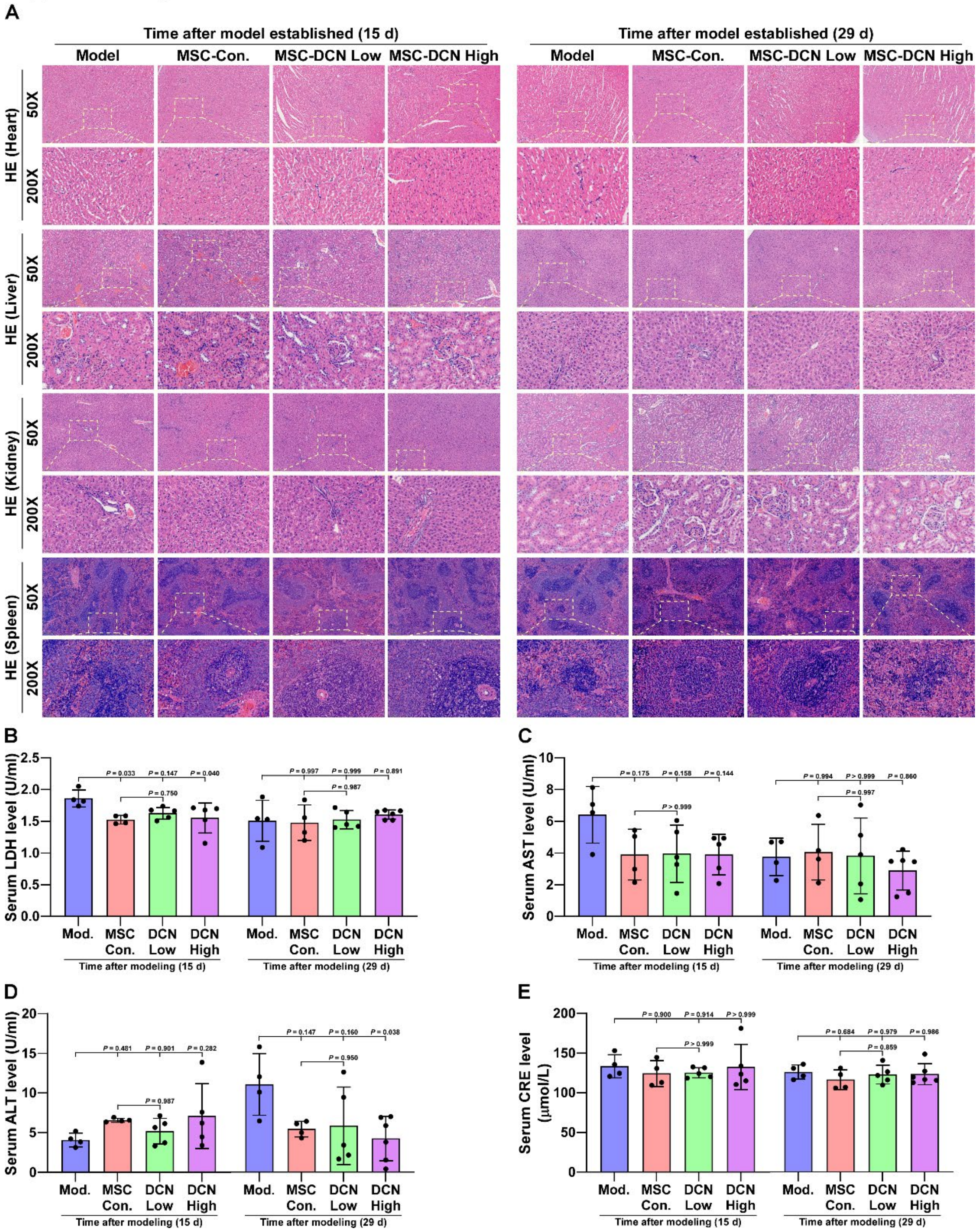

Supplemental figure 3. General reaction of Cell transplantation in Bleomycin-induced pulmonary fibrosis rat model treatment. (A) HE staining of heart, liver, kidney and spleen. (B) Serum LDH levels. (C) Serum AST levels. (D) Serum ALT levels. (E) Serum CRE levels. Each bar represents the mean± SD.

Supplemental figure 4

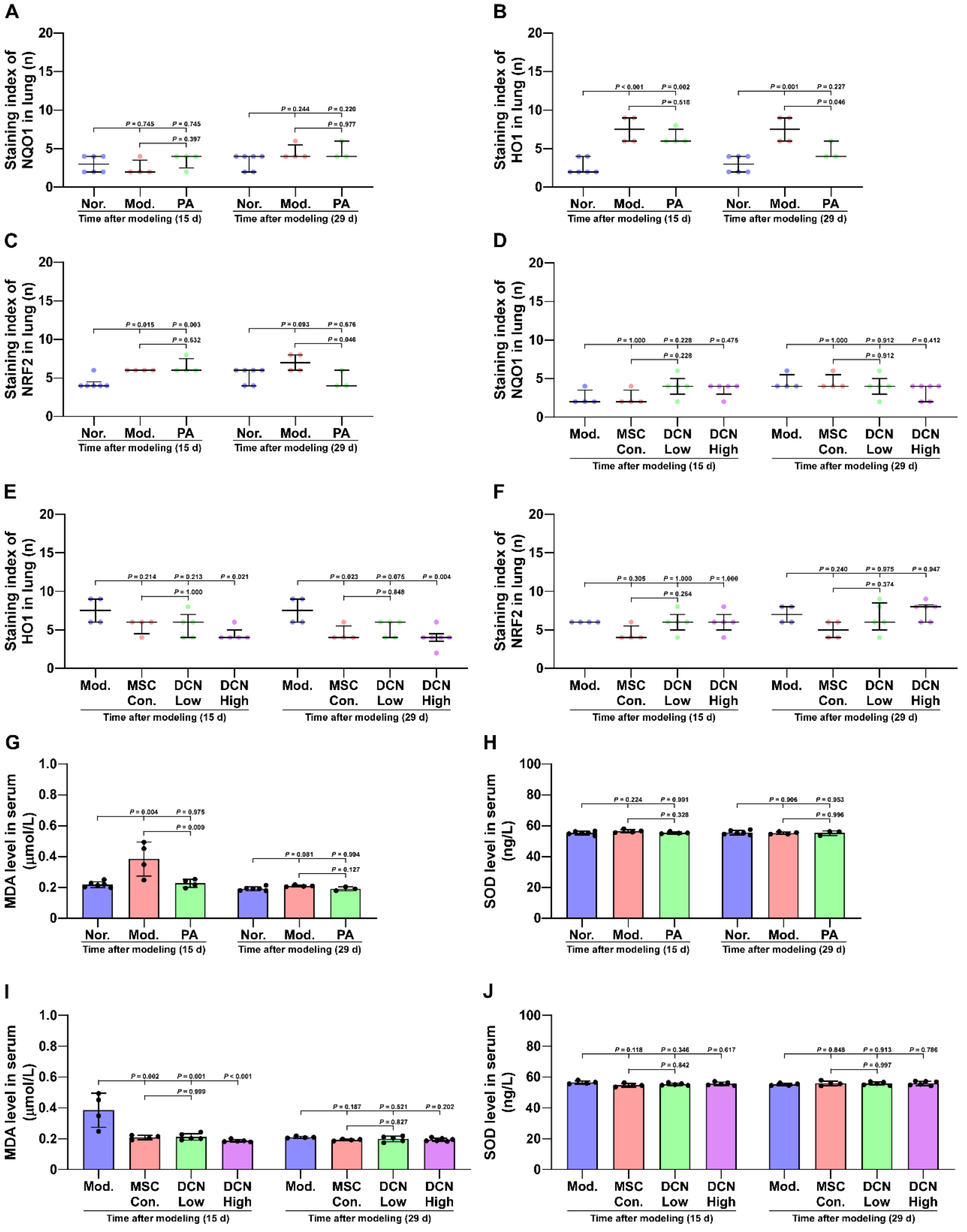

Supplemental figure 4. (A) Immunohistochemical staining index of NQO1 in lung tissue on days 15 and 29 after modelling. (B) Immunohistochemical staining index of HO1 in lung tissue on days 15 and 29 after modelling. (C) Immunohistochemical staining index of NRF2 in lung tissue on days 15 and 29 after modelling. (D) Immunohistochemical staining index of NQO1 in lung tissue on days 15 and 29 after modelling. (E) Immunohistochemical staining index of HO1 in lung tissue on days 15 and 29 after modelling. (F) Immunohistochemical staining index of NRF2 in lung tissue on days 15 and 29 after modelling. (G) Serum MDA levels. (H) Serum SOD levels. (I) Serum MDA levels. (J) Serum SOD levels. Each bar represents the mean± SD.

Supplemental figure 5

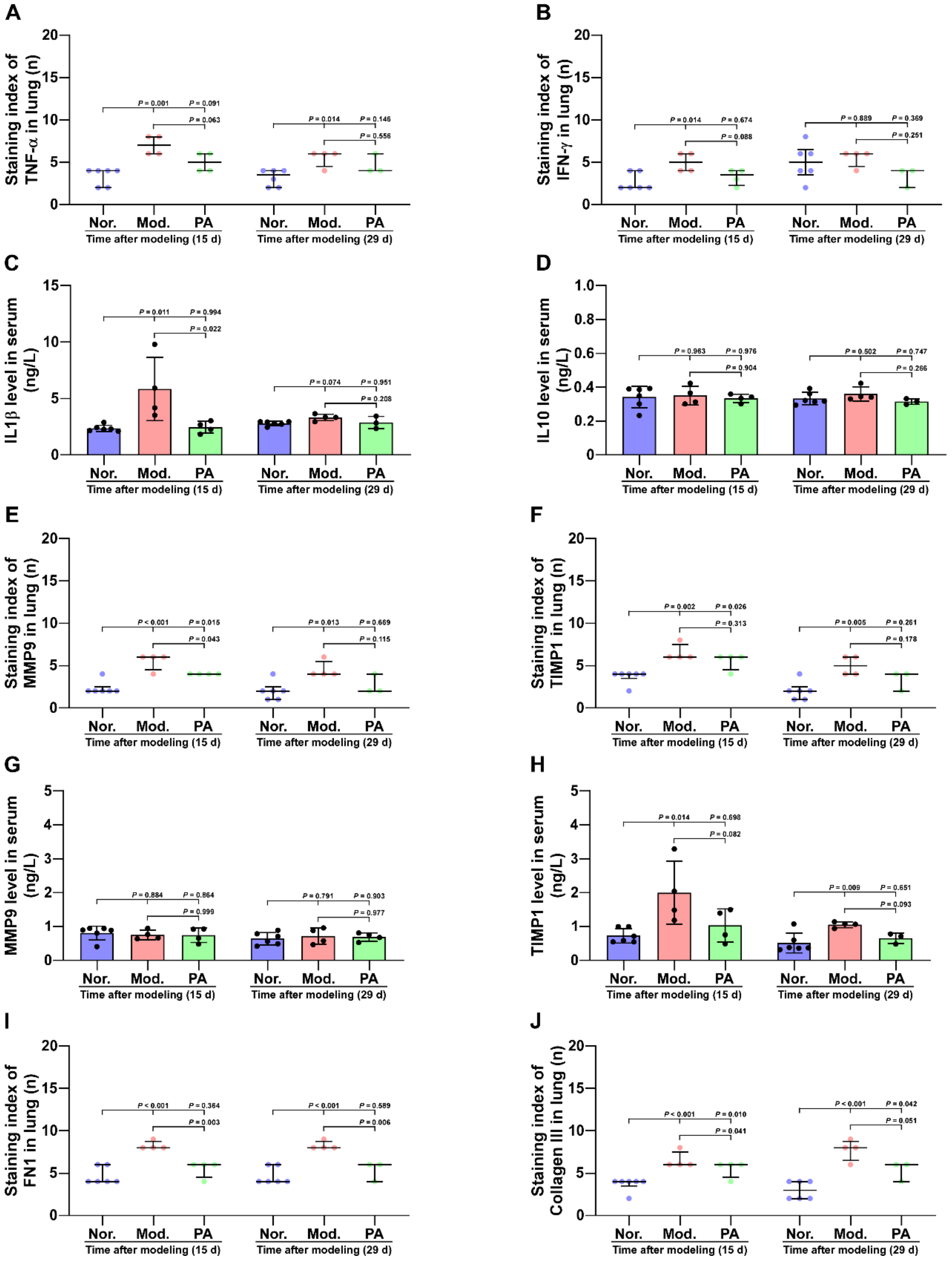

Supplemental figure 5. (A) Immunohistochemical staining index of TNF-α in lung tissue on days 15 and 29 after modelling. (B) Immunohistochemical staining index of IFN-γ in lung tissue on days 15 and 29 after modelling. (C) Serum IL1β levels. (D) Serum IL10 levels. (E) Immunohistochemical staining index of MMP9 in lung tissue on days 15 and 29 after modelling. (F) Immunohistochemical staining index of TIMP1 in lung tissue on days 15 and 29 after modelling. (G) Serum MMP9 levels. (H) Serum TIMP1 levels. (I) Immunohistochemical staining index of FN1 in lung tissue on days 15 and 29 after modelling. (J) Immunohistochemical staining index of COL

III in lung tissue on days 15 and 29 after modelling. Each bar represents the mean± SD.

Supplemental figure 6

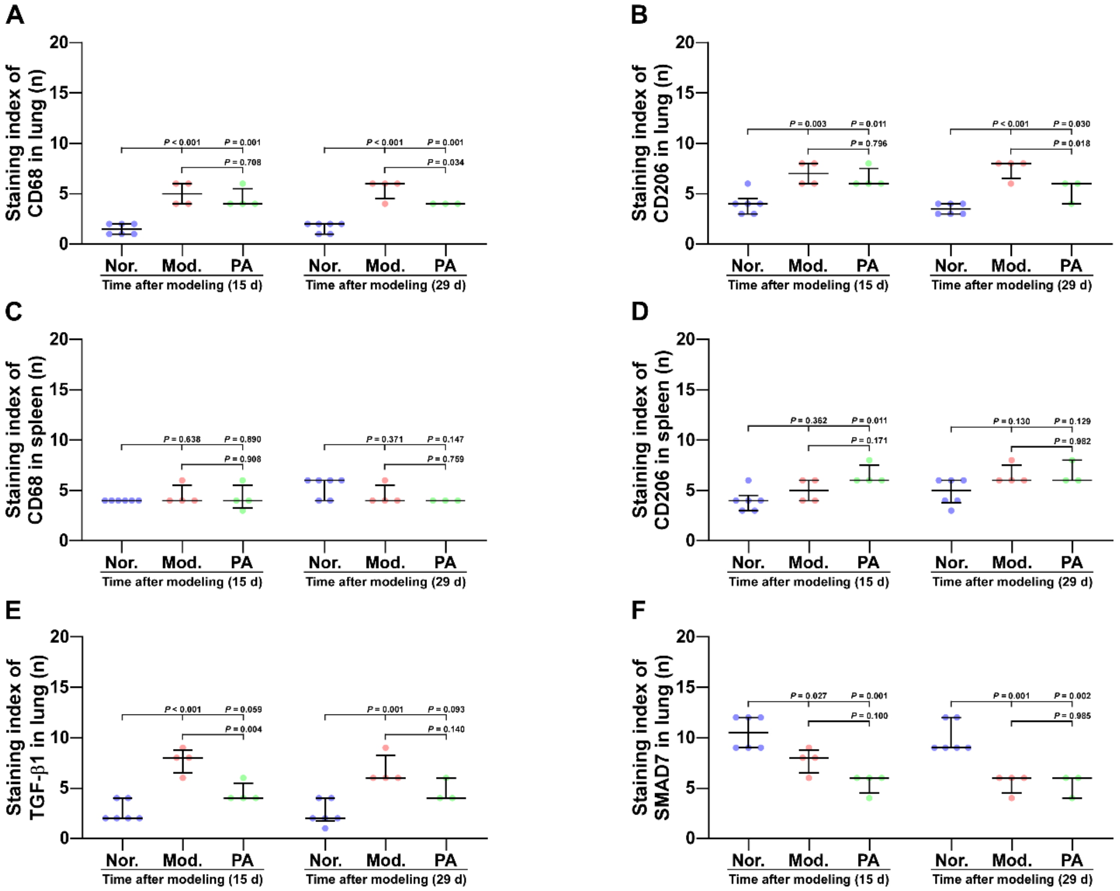

Supplemental figure 6. (A) Immunohistochemical staining index of CD68 in lung tissue on days 15 and 29 after modelling. (B) Immunohistochemical staining index of CD206 in lung tissue on days 15 and 29 after modelling. (C) Immunohistochemical staining index of CD68 in spleen tissue on days 15 and 29 after modelling. (D) Immunohistochemical staining index of CD206 in spleen tissue on days 15 and 29 after modelling. (E) Immunohistochemical staining index of TGF-β1 in lung tissue on days 15 and 29 after modelling. (F) Immunohistochemical staining index of Smad7 in lung tissue on days 15 and 29 after modelling.

Supplemental figure 7

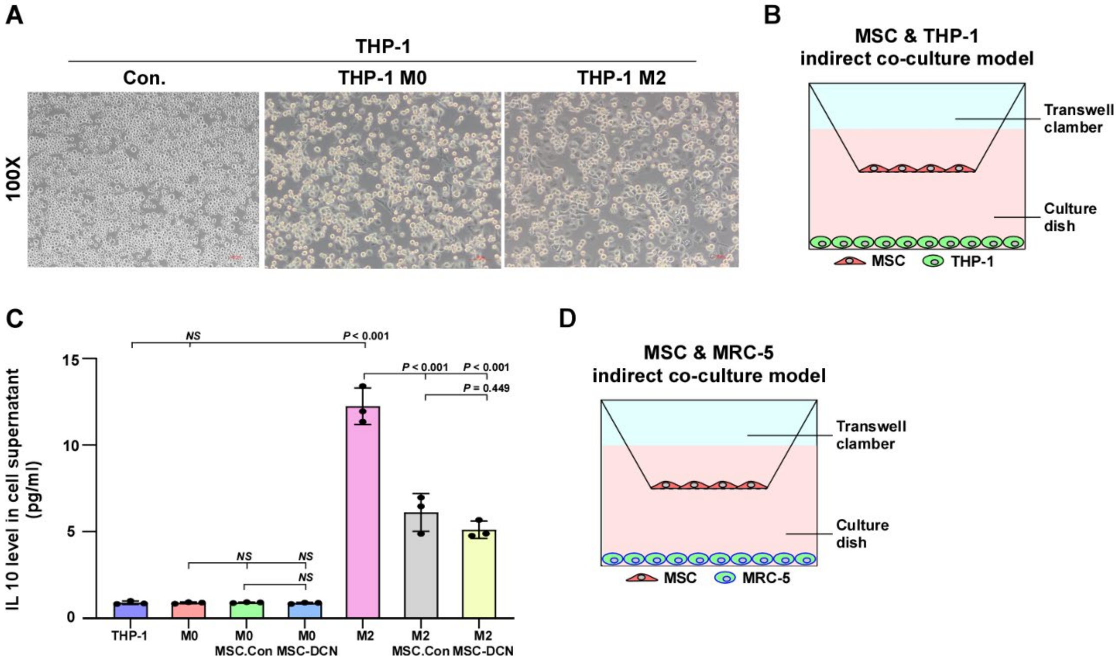

Supplemental figure 7. (A) Different states of THP-1. (B) Schematic diagram of MSCs and THP-1 cells co-culture. (C) IL10 levels in cell supernatant of different macrophages states. (D) Schematic diagram of MSCs and MRC-5 cells co-culture. Each bar represents the mean ± SD (n = 3).
